# Temporal-spatial changes in Sonic Hedgehog expression and signaling reveal different potentials of ventral mesencephalic progenitors to populate distinct ventral midbrain nuclei

**DOI:** 10.1186/1749-8104-6-29

**Published:** 2011-06-20

**Authors:** Sandra Blaess, Gabriela O Bodea, Anna Kabanova, Soline Chanet, Emilie Mugniery, Amin Derouiche, Daniel Stephen, Alexandra L Joyner

**Affiliations:** 1 Developmental Biology Program, Sloan-Kettering Institute, 1275 York Avenue, New York, NY 10021, USA; 2 Institute of Reconstructive Neurobiology, Life and Brain Center, University of Bonn, Sigmund-Freud-Str. 25, 53127 Bonn, Germany; 3 Institut Pasteur, GDD Unit, CNRS URA 2578, 25 rue du Dr Roux, 75015 Paris, France; 4 4Hôpital Necker - Enfants Malades, 149 rue de Sèvres - 75743 Paris cedex 15, France; 5 Institute of Cellular Neurosciences, University of Bonn, Sigmund-Freud-Str. 25, 53105 Bonn, Germany; 6 Institute for Anatomy II, Goethe-Universität Frankfurt, Theodor-Stern-Kai 7, 60590 Frankfurt am Main, Germany

## Abstract

**Background:**

The ventral midbrain contains a diverse array of neurons, including dopaminergic neurons of the ventral tegmental area (VTA) and substantia nigra (SN) and neurons of the red nucleus (RN). Dopaminergic and RN neurons have been shown to arise from ventral mesencephalic precursors that express *Sonic Hedgehog *(*Shh*). However, *Shh *expression, which is initially confined to the mesencephalic ventral midline, expands laterally and is then downregulated in the ventral midline. In contrast, expression of the Hedgehog target gene *Gli1 *initiates in the ventral midline prior to *Shh *expression, but after the onset of *Shh *expression it is expressed in precursors lateral to *Shh*-positive cells. Given these dynamic gene expression patterns, *Shh *and *Gli1 *expression could delineate different progenitor populations at distinct embryonic time points.

**Results:**

We employed genetic inducible fate mapping (GIFM) to investigate whether precursors that express *Shh *(Shh-GIFM) or transduce Shh signaling (Gli1-GIFM) at different time points give rise to different ventral midbrain cell types. We find that precursors restricted to the ventral midline are labeled at embryonic day (E)7.5 with Gli1-GIFM, and with Shh-GIFM at E8.5. These precursors give rise to all subtypes of midbrain dopaminergic neurons and the anterior RN. A broader domain of progenitors that includes the ventral midline is marked with Gli1-GIFM at E8.5 and with Shh-GIFM at E9.5; these fate-mapped cells also contribute to all midbrain dopaminergic subtypes and to the entire RN. In contrast, a lateral progenitor domain that is labeled with Gli1-GIFM at E9.5 and with Shh-GIFM at E11.5 has a markedly reduced potential to give rise to the RN and to SN dopaminergic neurons, and preferentially gives rise to the ventral-medial VTA. In addition, cells derived from *Shh*- and *Gli1*-expressing progenitors located outside of the ventral midline give rise to astrocytes.

**Conclusions:**

We define a ventral midbrain precursor map based on the timing of *Gli1 *and *Shh *expression, and suggest that the diversity of midbrain dopaminergic neurons is at least partially determined during their precursor stage when their medial-lateral position, differential gene expression and the time when they leave the ventricular zone influence their fate decisions.

## Background

The ventral mesencephalic progenitor domain generates a diverse array of distinct neuronal cell types, including neurons of the red nucleus (RN), motoneurons of the oculomotor nucleus and midbrain dopaminergic (DA) neurons. DA neurons are further organized into anatomically and functionally distinct subclasses [1]. The substantia nigra (SN), located in the lateral-ventral midbrain, projects to the dorsal-lateral striatum and is involved in the regulation of motor behaviors. The ventral tegmental area (VTA), located more medially, projects to corticolimbic targets and is important for motivational states. The retrorubral field is located posterior to the SN and projects to striatal, limbic and cortical areas. The functional diversity of these different regions becomes apparent in disease states: in Parkinson's disease, SN neurons, but not VTA neurons, degenerate, resulting in severe motor deficits. In contrast, abnormalities in the mesocorticolimbic system have been implicated in addiction, schizophrenia and attention deficit disorder [2-4]. While it is well established that the functional diversity of ventral midbrain neurons and DA subclasses is based on their distinct efferent and afferent connections and their distinct molecular make-up and physiology, it remains unclear when and how these distinct neuronal (sub)classes are established during development.

All midbrain DA neurons appear to arise from ventral mesencephalic floor plate progenitors that express *Sonic Hedgehog *(*Shh*) [5-8]. A recent paper utilizing genetic inducible fate mapping (GIFM) [9] suggested that *Shh *expression between embryonic day (E)7.5 and E12.5 sequentially marks three spatially distinct ventral mesencephalic progenitor domains that give rise to different neurons. However, the distribution of fate-mapped cells was only assessed qualitatively at embryonic stages, and a potential contribution to glia was not determined.

Gli1, a zinc finger transcription factor in the Shh signaling pathway, is only transcribed in cells that receive high levels of Hedgehog signaling (and are close to the source of Hedgehog) [10,11]; therefore, its expression can be used as a readout for cells that are exposed to high levels of Shh signaling [12]. *Shh*-expressing cells, including the floor plate cells themselves, do not respond to Shh signaling as measured by the expression of *Gli1 *[11-13]. It is therefore necessary to understand the exact timing of Shh responses and *Shh *expression in ventral midbrain precursors to gain a better insight into the role of Shh signaling in specification of ventral midbrain neurons.

To establish a precise precursor map of the ventral mesencephalon, we assessed the fate of *Gli1*-expressing (Shh-responding) and *Shh*-expressing progenitors with GIFM in a quantitative manner at embryonic and postnatal stages. We show that *Gli1 *expression precedes *Shh *expression by about a day and demonstrate that ventral midbrain precursors that give rise to DA neurons respond to Shh signaling between E7.5 and E9.5 and express *Shh *between E8.5 and E11.5. Progenitors in the ventral midline that are labeled with Gli1-GIFM at E7.5 and with Shh-GIFM at E8.0 to E8.5 contribute to midbrain DA neurons and the anterior RN. Progenitors in a broader domain are marked with Gli1-GIFM at E8.5 and Shh-GIFM at E9.5 to E10.5 and show a strong contribution to all subsets of DA neurons and to RN neurons. Precursors adjacent to the ventral midline that are fate-mapped with Gli1-GIFM at E9.5 and Shh-GIFM at E11.5 maintain the potential to develop into DA neurons of the ventral-medial VTA. However, they contribute few cells to DA neurons in the SN and to RN neurons. In addition, precursors labeled with Gli1-GIFM at E8.5 to E9.5 give rise to other ventral midbrain neurons, including neurons in the oculomotor nucleus and the non-DA neurons in the SN reticularis, consistent with a broad medial-lateral distribution of *Gli1*-expressing precursors. Finally, we observe that *Shh*- and *Gli1*-expressing progenitors, with the exception of progenitors in the ventral midline, develop into ventral midbrain astrocytes.

## Materials and methods

### Fate mapping

Animal studies were performed under an approved Institutional Animal Care and Use Committee animal protocol according to the institutional guidelines at Memorial-Sloan Kettering Cancer Center or were approved by the University of Bonn Animal Care and Use Committee. *ROSA*^*loxP-STOP-loxP-LacZ *^(*R26*^*lz*^) reporter mice were kindly provided by Dr P Soriano [14]; *ROSA*^*loxP-STOP-loxP-EYFP *^(*R26*^*EYFP*^) mice were kindly provided by Dr F Costantini [15]; *Shh*^*CreERneo *^mice (previously called *Shh*^*CreERT2 *^[9,16]) were kindly provided by Dr C Tabin. The *neo *cassette in the *Shh*^*CreERneo *^allele was deleted with an *ACTB-Flpe *deleter line [17] and only mice without the *neo *cassette (referred to as *Shh*^*CreER *^mice) were used for fate mapping experiments. The *Gli1*^*CreER *^mice without *neo *were described previously [12]. Mice heterozygous for the *R26*^*lz *^or *R26*^*EYFP *^allele and the *CreER *alleles were genotyped as previously described [14,18]. All mice were maintained in an outbred SW or CD1 background. *Shh*^*CreER*^^/^^*+*^*R26*^*lz*^^/^^*lz*^, *Shh*^*CreER*^^/^^*+*^*R26*^*EYFP*^^/^^*EYFP*^, *Gli1*^*CreER*^^/+^*R26*^*lz*^^/^^*lz *^or *Gli1*^*CreER*^^/+^*R26*^*EYFP*^^/^^*EYFP *^males were bred with SW or CD1 wild-type females (Taconic, Hudson, NY, USA or Charles River, Wilmington, MA, USA) to generate *Shh*^*CreER*^^/^^*+*^*R26*^*lz*^^/^^*+*^, *Shh*^*CreER*^^/^^*+*^*R26*^*EYFP*^^/^^*+*^, *Gli1*^*CreER*^^/+^*R26*^*lz*^^/^^*+ *^or *Gli1*^*CreER*^^/+^*R26*^*YFP*^^/^^*+ *^progeny. Noon of the day that a vaginal plug was detected was designated as E0.5. Tamoxifen (TM; T-5648 Sigma, St. Louis MO, USA) was dissolved in corn oil (Sigma C-8267) at a final concentration of 20 mg/ml. Pregnant females were given 3 to 4 mg TM through oral gavage with animal feeding needles (Fisher Scientific, Waltham, MA, USA and Fine Science Tools, Heidelberg, Germany) at 12 pm for GIFM between E6.5 and E12.5 or at midnight for GIFM at E8.0. For the analysis of fate-mapped cells at postnatal stages, the TM solution contained progesterone (Sigma P-0130) at a concentration of 5 mg/ml to reduce the incidence of miscarriages.

### Tissue processing, RNA *in situ *hybridization and immunofluorescence stainings

Embryos or embryonic brains were dissected and fixed in 4% paraformaldehyde for 20 to 90 minutes. Postnatal day (P)14 to P60 mice were perfused intracardially with 4% paraformaldehyde, and brains were dissected and postfixed in 4% paraformaldehyde overnight. E8.5 to E14.5 embryos or embryonic brains were sectioned on a cryostat at 12 μm, E18.5 brains at 14 μm and postnatal brains at 40 μm (free-floating sections). For RNA *in situ *hybridization, frozen sections were used or the tissue was processed manually or in a Leica tissue processor for paraffin embedding and sectioned at 7 μm. RNA *in situ *hybridization was performed as described [19]. X-gal and immunofluorescent stainings were performed using standard procedures [19]. Primary antibodies: goat anti-β-galactosidase (β-gal; 1:2,000, AbD Serotec, Oxford, UK), rabbit or rat anti-GFP (1:400, Invitrogen, Carlsbad, CA, USA or 1:2,000, Nacalai, Kyoto, Japan) rabbit or mouse anti-tyrosine hydroxylase (TH; 1:500, Millipore, Billerica, MA, USA), rabbit anti-Calbindin (1:5,000, Swant, Marly, Switzerland), rabbit anti-Lmx1a (1:2,000, Millipore), mouse anti-Pou4f1 (1:100, Santa Cruz Antibodies, Santa Cruz, CA, USA), rabbit anti-glial fibrillary acidic protein (GFAP; 1:500, Millipore), mouse anti-glutamine synthetase (1:500, Millipore) and rabbit anti-Girk2 (1:100, Alomone Labs, Jerusalem, Israel). The mouse anti-Islet1 (Isl1, 1:50) and anti Nkx2-2 antibody (1:50) developed by T Jessell and S Brenner-Morton and the mouse anti-Nkx6-1 antibody (1:100) developed by OD Madsen were obtained from the Developmental Studies Hybridoma Bank. For Brn3a, Isl1, Lmx1a, Nkx2-2 and Nkx6-1, sections were incubated in 0.1 mM EDTA for 10 minutes at 65°C prior to the immunostainings. Secondary antibodies: donkey anti-goat IgG-Alexa 555 and donkey anti-rabbit IgG-Alexa 488 (1:500; Invitrogen); donkey anti-goat Cy3, donkey anti-rabbit Cy3 or fluorescein isothiocyanate (FITC), donkey anti-mouse Cy3 or FITC (1:200, Jackson ImmunoResearch, West Grove, PA, USA). Isl1, Nkx2-2 and Nkx6-1 were detected with donkey-anti mouse biotin secondary antibody (1:200, Jackson ImmunoResearch), followed by Cy3- or dichlorotriazinylaminofluorescein (DTAF)-labeled streptavidin (1:1,000, Jackson ImmunoResearch).

### Quantification

For the quantification of fate-mapped cells at E18.5, at a minimum every tenth section was stained with β-gal antibody and antibodies against markers of ventral midbrain neurons (TH, Pou4f1, Isl1). Sections containing midbrain DA, RN or motoneurons were selected and the entire area containing the neurons of interest was photographed using a 20 × objective. To assess double labeling, Z-stacks were taken and subjected to deconvolution (Volocity Software, Perkin Elmer, Waltham, USA). To cover the entire area, single pictures were taken on a Leica DM6000 microscope (Leica Microsystems, Wetzlar, Germany) and stitched together in Photoshop (Adobe Systems Inc.). Alternatively, a single optical section of the area was imaged with a Zeiss Axio observer microscope using Mosaix software and an Apotome setup (Axiovision, Zeiss, Oberkochen, Germany). Cells positive for both the neuronal marker and β-gal were counted. Sections were counted from at least three animals. At least four (TH) or at least three sections (Pou4f1) were counted per animal. The average number of double-labeled cells per section was determined for each animal. The values presented are the average values of three to five animals. Significance (at *P *< 0.05) was determined using Student's *t*-test. For the regional quantification, sections from four rostral-caudal DA-neuron-containing midbrain regions were selected based on the anatomy of the TH-positive nuclei in the ventral midbrain. The number of cells in each region was normalized for the number of sections counted. The values presented are the average values of at least three animals. Significance (at *P *< 0.05) was determined by analysis of variance (ANOVA) and least significant difference (LSD) post-hoc analysis.

For the quantification of the overall distribution of DA neurons and of DA neurons labeled with Shh- or Gli1-GIFM in postnatal brains, sections at four rostral-caudal levels (approximately Bregma -2.92, Bregma -3.18, Bregma -3.40, Bregma -3.64) were picked from at least three animals for each time point. Sections were imaged on a Zeiss Axio observer using the Zeiss Mosaix software and an Apotome setup to assess double labeling (Axiovision, Zeiss). For each level, the areas containing TH-positive DA neurons were outlined [20] and the number of β-gal- and TH-positive or enhanced yellow fluorescent protein (EYFP)- and TH-positive cells located in the SN, dorsal-lateral VTA (dlVTA) and ventral-medial VTA (vmVTA) was determined. If more than one section was counted per level, numbers were averaged for the number of evaluated sections. Significance (at *P *< 0.05) was determined by ANOVA and LSD post-hoc analysis or Student's *t*-test.

## Results

### The *Shh *expression domain in the ventral mesencephalon undergoes a medial to lateral shift during embryonic development

**Figure 1:**
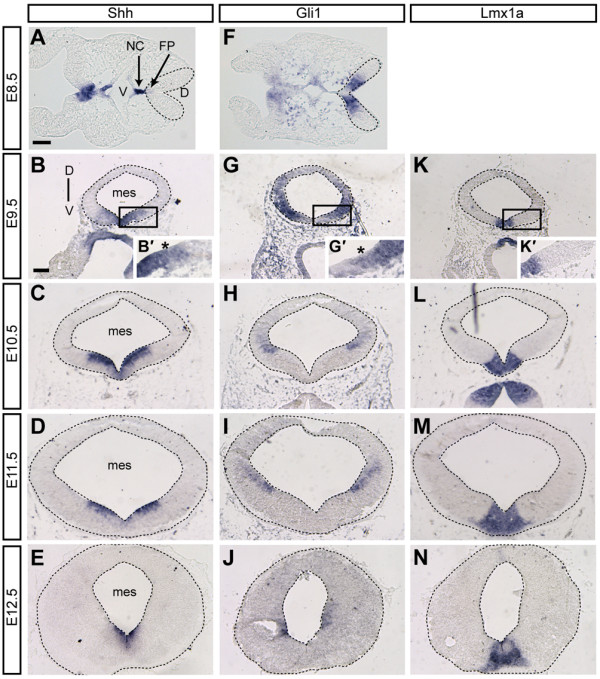
**Dynamic changes in *Shh *and *Gli1 *expression in the ventral mesencephalic neural tube**. RNA *in situ *hybridization with *Shh*, *Gli1 *and *Lmx1a *RNA probes. The analysis was performed on transverse sections (E8.5) or coronal sections of the mesencephalon (mes; E9.5 to E12.5); sections shown are at the level of the intermediate mes. V, ventral; D, dorsal. The mesencephalon is outlined. **(A-E) ***Shh *is initially expressed in the notochord (NC) and is induced in the mesencephalon floor plate (FP) at E8.5. *Shh *expression expands laterally over the subsequent days of development (E9.5 to E12.5), but is downregulated medially (E10.5 to E12.5). **(F-J) **At all time points analyzed, *Gli1 *expression, a readout for high levels of Shh signaling, is located laterally to the *Shh-*expressing cells. *Gli1 *expression is largely absent in the *Shh*-expressing domain, indicating that *Shh-*expressing cells do not respond to high level Shh signaling. At E9.5, the weak ventral expression domain of *Gli1 *and the weak lateral expression domain of *Shh *appear to overlap partially (B',G', asterisks), but at later stages, the *Shh *and *Gli1 *domains are clearly separated. Note that *Gli1 *expression is not upregulated in medial cells that downregulate *Shh *expression. **(K-N) ***Lmx1a *is expressed in the DA precursor domain and in differentiating DA neurons. Scale bars: (A,F) 50 μm; (B-E,G-J,K-N) 100 μm.

We and others have previously shown that *Shh *expression is dynamic in the ventral midbrain [8,13,21-23]. We investigated the temporally dynamic expression pattern of *Shh *in the ventral midbrain precursor domain in more detail by performing *in situ *hybridization analysis of *Shh *mRNA on transverse (E8.5) or coronal sections (all other stages) of embryos each day between E8.5 and E12.5. As previously described, we found that at E8.5, *Shh *expression was restricted to a narrow medial domain overlying the notochord, and the domain expanded laterally until E10.5, when *Shh *expression began to be downregulated in the medial domain (Figure 1A-E). However, in contrast to previous studies [8,23], weak Shh expression was detected in the medial domain at E11.5 and E12.5. In comparison to *Shh*, the expression of *Lmx1a*, a putative marker of the DA precursor domain, which is first expressed in the ventral midbrain at E9.0, was found to have a less dynamic expression pattern and was restricted to a more medial domain [23] (Figure 1K-N).

### The location of *Gli1*-expressing (Shh-responding) ventral mesencephalic precursors shifts laterally over time

*Gli1 *is initially expressed in the ventral midline of the neural tube at E7.5 [24], suggesting that the ventral mesencephalic neural tube receives high levels of Shh signaling from the notochord at E7.5. We observed that once *Shh *expression was present in ventral midline cells (starting around E8.5), *Gli1 *expression was downregulated in the *Shh-*expressing cells and was excluded from the midline, indicating that *Shh*-expressing cells cease responding to Shh signaling (Figure 1F-J) [13,22]. At E8.5 and subsequent stages, *Gli1 *was expressed in precursors adjacent to the *Shh*-expressing domain (Figure 1F-J). At E9.5, there was some overlap between *Shh *and *Gli1 *expression at their domain boundaries, where both *Gli1 *and *Shh *were expressed at low levels and/or in a mosaic manner (Figure 1B',G' and data not shown). At later stages the *Gli1 *and *Shh *expression domains appeared to be clearly separated (Figure 1C,H,D,I,E,J). Interestingly, *Gli1 *expression was not induced in the medial domain after *Shh *expression was downregulated at E10.5, perhaps because a low level of *Shh *expression remained. In summary, we demonstrate that there is a medial to lateral shift in the *Shh *and *Gli1 *expression domains between E8.5 and E12.5 in the ventral mesencephalon. *Gli1 *expression is found lateral to the *Shh*-expressing domain at all time points. Thus, the time period when *Shh *and *Gli1 *are expressed in spatially defined progenitor domains could potentially delineate progenitor populations of different ventral midbrain cell types.

### Fate mapping strategy to follow the fate of progenitors in different medial-lateral *Shh*- and *Gli1*-expressing mesencephalic domains

**Figure 2:**
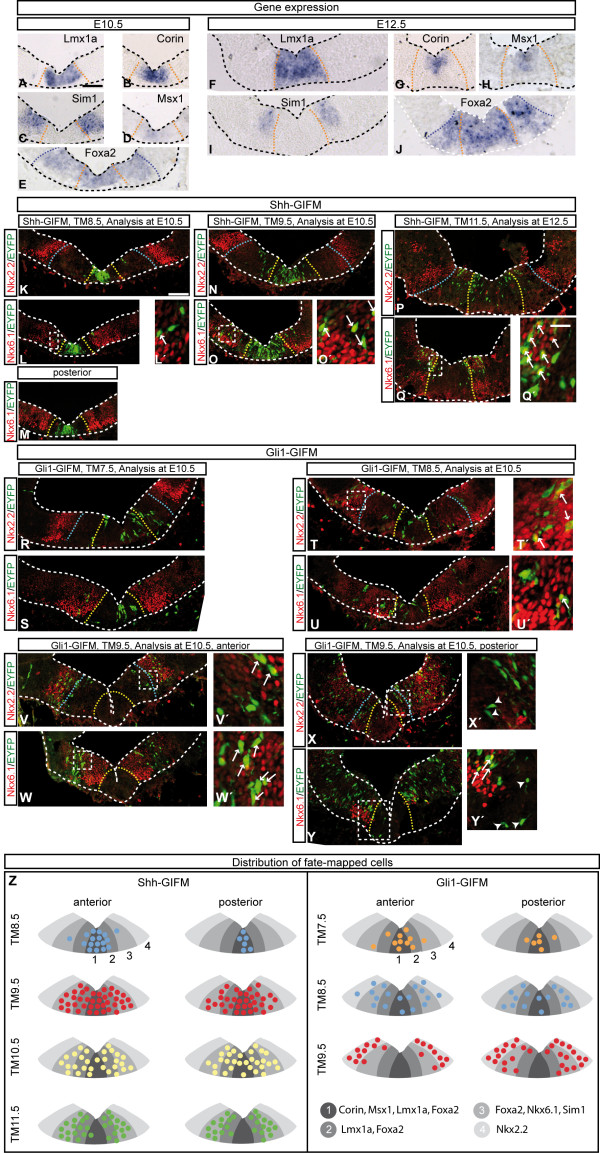
**Initial domains of cells marked with Shh- or Gli1-GIFM in comparison with other ventral midbrain markers**. **(A-Y) ***In situ *hybridization (A-J) and immunostainings (K-Y) on E10.5 and E12.5 coronal sections for markers of the DA precursor domain (*Lmx1a*, *Msx1*, *Corin*) and the RN precursor domain (*Sim1*, Nkx6-1). *Foxa2 *encompasses both precursor domains. Nkx2-2 labels a precursor domain that is thought to give rise to GABAergic interneurons. (K-Y) Shh-GIFM (K-Q') and Gli1-GIFM (R-Y'). TM was administered at the indicated time points and marked cells were analyzed at E10.5 or E12.5 with EYFP (green) and Nkx6-1 or Nkx2-2 (red) immunostaining. The *Lmx1a-*expressing (yellow or orange dashed line) and the *Foxa2*-expressing (blue dashed lines) cells are outlined in some sections. Arrows indicate double-labeled cells, arrowheads in X' and Y' indicate fate-mapped cells in the Nkx6-1 negative medial domain. The medial-lateral extent of the initial fate-mapped domains reflects the endogenous gene expression around the time of TM administration (compare with Figure 1). Note that the labeling is mosaic, since only a subset of cells is recombined in a given domain. Panels (L'-Y') are higher magnifications of the areas indicated with the dashed box in (L-Y). **(Z) **Distribution of cells fate-mapped at the indicated time points. The summary is based on the immunostainings and *in situ *hybridizations at E10.5 and E12.5. To assess the distribution of the fate-mapped cells, at least three sections were analyzed for each TM time point at E10.5 and E12.5. To determine the expression domains of the specific transcription factors, sections from at least three embryos were analyzed. Scale bars: 100 μm.

**Figure 3:**
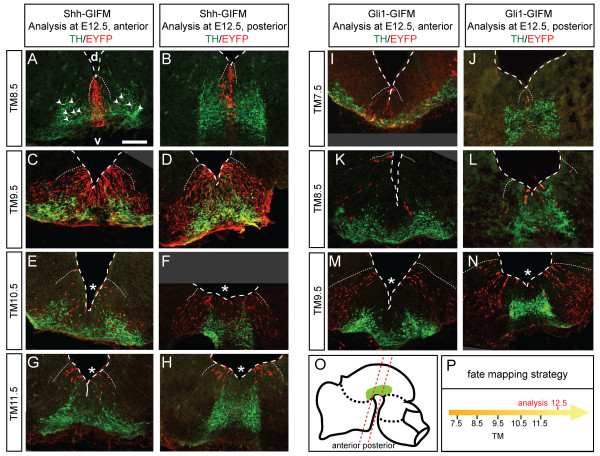
**Changing populations of precursors are marked with Shh- and Gli1-GIFM at different stages of development**. **(A-N) **Immunofluorescent staining for DA neurons (TH, green) and EYFP-positive fate-mapped cells (red) on E12.5 coronal sections of the mesencephalon showing the distribution of marked cells in the ventricular zone and their contribution to TH-positive DA neurons. The labeling is mosaic, since only a subset of cells is recombined in a given domain. Asterisks indicate a decreased contribution of marked cells to the medial precursor domain. Arrowheads in (A) indicate TH/EYFP-positive cells that are located more lateral than their precursor domain. The mesencephalon ventricle is outlined. v, ventral; d, dorsal. Note that there is less recombination with Gli1-GIFM (I-N) than with Shh-GIFM (A-K). Scale bar: 100 μm. **(O) **The representative sections shown in (A-N) are at the level of the anterior and posterior mesencephalon as indicated in the schematic. **(P) **Schematic of fate mapping strategy.

To determine whether precursors in the temporally different *Shh*- or *Gli1*-positive domains give rise to distinct (sub)classes of ventral midbrain neurons, we used GIFM [25]. This technique provides temporal and spatial control of cell marking by utilizing an inducible form of site-specific recombinase, CreER and a reporter allele, which permanently expresses a marker gene (for example, lacZ, EYFP) after Cre-mediated recombination [26]. Temporal control of marking is achieved by administering TM at specific time points in development to activate CreER. For our GIFM approach, we used mouse lines in which a TM-inducible form of Cre (CreERT2) is expressed by the *Shh *or *Gli1 *allele (*Shh*^*CreER *^or *Gli1*^*CreER *^line) [12,16]. As nuclear translocation of CreER occurs within 6 hours of TM administration and is maintained for approximately 24 hours [22,27,28] cells expressing *Shh *and *Gli1 *at 6 to 36 hours after TM administration can be genetically marked. The ubiquitously expressed *ROSA*^*loxP-STOP-loxP-LacZ *^(*R26*^*lz*^) or *ROSA*^*loxP-STOP-loxP-EYFP *^(*R26*^*EYFP*^) alleles [14,15] were used to permanently mark *Shh*- or *Gli1*-expressing precursors at distinct developmental time points and to track the fate of cells derived from the genetically marked precursors. We refer to the time point of TM injection as TM followed by the embryonic day (for example, TM administration at E8.5 is TM8.5). X-gal staining or immunohistochemistry for β-gal 24 hours after TM administration showed that the resultant marking was mosaic (less than 100% of cells expressing *Shh *or *Gli1 *were marked; compare Figures 1 and 2; data not shown). In an initial GIFM study with the original *Shh*^*CreERneo *^allele that contains an FRT-flanked *neo *cassette downstream of CreER [16], we observed only a few lacZ-positive cells in each section of the mesencephalon when administering 3 to 4 mg of TM (Additional file 1A-C and data not shown), making it very difficult to investigate the fate of these cells in a quantitative manner. Since we previously found that a *neo *cassette can decrease CreER expression in knock-in alleles with a similar design [29], we removed the *neo *cassette with an *ACTB-Flpe *deleter line [17]. Using the new line (*Shh*^*CreER*^) for GIFM with the same dose of TM, we found indeed many more lacZ-positive cells in the mesencephalon (Additional file 1 and data not shown). In general, we observed more overall recombination with the *Shh*^*CreER *^line than with the *Gli1*^*CreER *^line (Figures 2 and 3; Additional file 2). The lower recombination efficiency of the *Gli1*^*CreER *^line is likely due to overall lower expression levels of *Gli1 *(and therefore *CreER*). Alternatively, *Gli1 *could be expressed at different levels in subsets of cells with only some cells expressing high enough levels of *CreER *to induce recombination. Such a bias of GIFM towards marking of cells with higher expression levels is inherent to the technique. The spatial limits of the domains of mosaic marking in animals that received TM at the same time point and were analyzed at the same developmental stage were very similar between animals for each *Crier *allele, with variations only in the percentage of cells marked (data not shown).

### *Shh *and *Gli1 *expression define distinct precursor domains in the ventral mesencephalon

As a first step in determining the fate of *Shh*- and *Gli1-*expressing ventral midbrain progenitors, we confirmed that the fate-mapped domains corresponded to the observed mRNA expression patterns of *Shh *and *Gli1*. In addition, we assessed how the distribution of the *Shh*- and *Gli1*-expressing precursor relates to other ventral mesencephalic precursor markers. To this end, *Shh*- and *Gli1*-expressing precursor cells marked at distinct time points (between E7.5 and E12.5 for Shh-GIFM and between E6.5 and E9.5 for Gli1-GIFM) were analyzed in the embryonic ventral mesencephalon at E12.5, and at E9.5 and E10.5 where applicable (Figures 2 and 3 and data not shown). The distribution of fate-mapped cells was compared with the expression of known ventral midline markers using either immunofluorescence staining for EYFP and the relevant marker or RNA *in situ *hybridization on adjacent sections (Figure 2 and data not shown; Additional file 2). Lmx1a, Corin and Msx1 are putative markers for the DA precursor domain, but Msx1 and Corin appear to be more medially restricted than Lmx1a [23,30]. Nkx6-1 and Sim1 are putative markers for precursors of the RN and motoneurons. Foxa2 is expressed in the Lmx1a- and Nkx6-1-positive domains. Nkx2-2 is a putative marker for precursors of GABAergic neurons [23,31,32] (Figure 2; Additional file 3). To identify the nascent DA region at E12.5, β-gal immunostaining for fate-mapped cells was combined with staining for TH, a marker for DA neurons (Figure 3) [33].

Shh-GIFM with TM7.5 resulted in the labeling of cells in the midline, but only in the anterior-most mesencephalon (data not shown). When marked with TM8.5 and analyzed at E9.5 and E10.5, cells derived from *Shh*-expressing progenitors (hereafter referred to as Shh-derived cells) were restricted to a narrow medial progenitor domain nested within the *Msx1*/*Corin*/*Lmx1a*/*Foxa2*-positive domain, with only a few anterior cells overlapping with Nkx6-1 (n = 4; Figures 2A,B,D,K-M and 3A,B and data not shown; Additional file 2I). Cells marked with TM9.5 and analyzed at E10.5 or E12.5 were distributed over a broader ventral domain that was nested within the *Foxa2*-positive domain and spanned the *Lmx1a*/*Msx1*/*Corin *as well as most of the Nkx6-1/*Sim1*-positive domains (n = 3; Figures 2A-E,N,O and 3C,D and data not shown; Additional file 2A,B,I). At E10.5, the domain labeled with Shh-GIFM at E9.5 appeared to be more medially restricted than at E12.5. This could be due to an incomplete recombination of the reporter allele at E10.5 (24 hours after TM administration). The medial-lateral extent of Shh-derived cells was maintained with TM10.5, but fewer cells were observed medially (n = 3; Figure 3E,F and data not shown). With TM11.5 (analyzed at E12.5) and TM12.5 (analyzed at E13.5) only the more lateral cells were labeled. These lateral precursors were located in the Nkx6-1/*Sim1*/*Foxa2 *expressing domain and in the lateral aspects of the *Lmx1a*-positive domain (n = 3; Figures 2F-J,P,Q and 3G,H and data not shown; Additional file 2C,D,I). Since we observed weak medial expression of *Shh *in our gene expression analysis at E11.5 and E12.5 (Figure 1D,E), the lack of medial labeling is likely due to CreER expression levels being too low to induce recombination of the reporter allele. The medial-lateral extent of the domains changed only slightly along the anterior-posterior axis of the developing mesencephalon, except for fate mapping with TM8.5 when the medial domain was even more narrowly restricted in posterior areas (Figure 3A,B). Finally, analysis at E12.5 showed that *Shh*-expressing progenitors marked with GIFM between E8.5 and E11.5 overlapped with TH expressing cells (Figure 3A-H and data not shown; Additional file 2J).

GIFM of *Gli1*-expressing cells resulted in sparser labeling than Shh-GIFM, indicating that only a small number of cells that express *Gli1 *undergo recombination (compare Figure 2T and Figure 1F). Nevertheless, analysis of several animals for each induction time point allowed us to gain insight into the distribution of the fate-mapped cells. Consistent with the mRNA expression pattern of *Gli1*, we found that initial marking of Gli1-derived cells with TM6.5 produced a small number of labeled cells in the ventral mesencephalic progenitor zone (one to two cells per section) in a minority of embryos (three of nine) (data not shown). With TM7.5, Gli1-derived cells were mainly localized in a narrow medial domain (Figures 2R,S and 3I,J), similar to the domain occupied by Shh-derived cells labeled with TM8.5, but a few cells were also found in the adjacent Nkx6-1 domain (n = 3; Figure 2R,S). With TM8.5, the Gli1-derived cells covered a broader ventral domain (Figures 2T,U and 3K,L; Additional file 2E,F,K), with less cells observed medially and some marked cells located in the Nkx2-2-positive domain (n = 4; Figure 2T,U). With TM9.5, Gli1-derived cells were located in a lateral domain (Figures 2V-Y and 3M,N; Additional file 2G,H,K) that overlapped with the Nkx2-2-positive domain and the lateral edges of the *Lmx1a*/*Foxa2*-positive domain in posterior sections. Marked cells were largely excluded from the *Lmx1a*-expressing domain in the anterior midbrain (n = 3; Figures 2A-E,V,W and 3M; Additional file 2G,K).

The analysis of Shh- and Gli1-derived precursors at E12.5 allowed us to assess whether the dynamic changes in the *Shh *and *Gli1 *gene expression domain were due to lateral expansion of proliferating precursor cells or to new populations of precursors expressing *Shh *and *Gli1 *at different stages of development. If the changes in *Shh *expression are due to an expansion of cells initially located medially, fate mapping of cells marked at early (E8.5, medial domain) or later (E9.5, broad domain, and E11.5, lateral domain) time points should result in identical progenitor domains at E12.5. If instead the changes in gene expression are due to more laterally located progenitor cells gradually switching on *Shh *or *Gli1 *expression (and medially located precursors switching off *Shh *or *Gli1 *expression), distinct domains should be seen at E12.5 following TM administration at different time points. In line with the second model, the fate-mapped (EYFP-positive) Shh-derived progenitors in the E12.5 ventral neural tube were located in distinct medial-lateral domains depending on the time point of TM administration. This was also the case for Gli1-derived cells (Figure 3).

In summary, our analysis at several embryonic time points shows that our GIFM approach faithfully marks *Shh*- and *Gli1*-expressing progenitor domains. Furthermore, the data demonstrate that during ventral midbrain development, *Gli1 *expression precedes *Shh *expression and that medial and medial-lateral ventral precursors that initially express *Gli1 *switch to expressing *Shh*. In addition, we demonstrate that on each embryonic day between E7 and E13, spatially distinct domains of precursors can be marked with Shh- or Gli1-GIFM (Figures 2 and 3). Four progenitor domains can be defined in the ventral midbrain based on combinations of gene expression. They correlate in the following manner with the *Shh *and *Gli1 *fate map (Figure 2Z; Additional file 3): a medial domain (domain 1) positive for *Lmx1a*, *Foxa2*, *Corin *and *Msx1 *that is marked with Gli1-GIFM at E7.5 to E8.5 and with Shh-GIFM at E8.5 to E10.5; a para-medial domain (domain 2) positive for *Lmx1a *and *Foxa2 *that is sequentially labeled with Gli1-GIFM (at E8.5 and in posterior sections also at E9.5) and with Shh-GIFM (E9.5 to E12.5); a medial-lateral domain (domain 3) positive for *Foxa2*, *Sim1 *and Nkx6-1 that is first labeled with Gli1-GIFM (at E8.5 and in posterior sections also at E9.5) and then with Shh-GIFM (E9.5 to E12.5); and a Nkx2-2-positive lateral domain (domain 4) that is only marked with Gli1-GIFM after E8.5.

### Progenitors expressing *Shh *between E8.5 and E12.5 give rise to dopaminergic and red nucleus neurons

**Figure 4:**
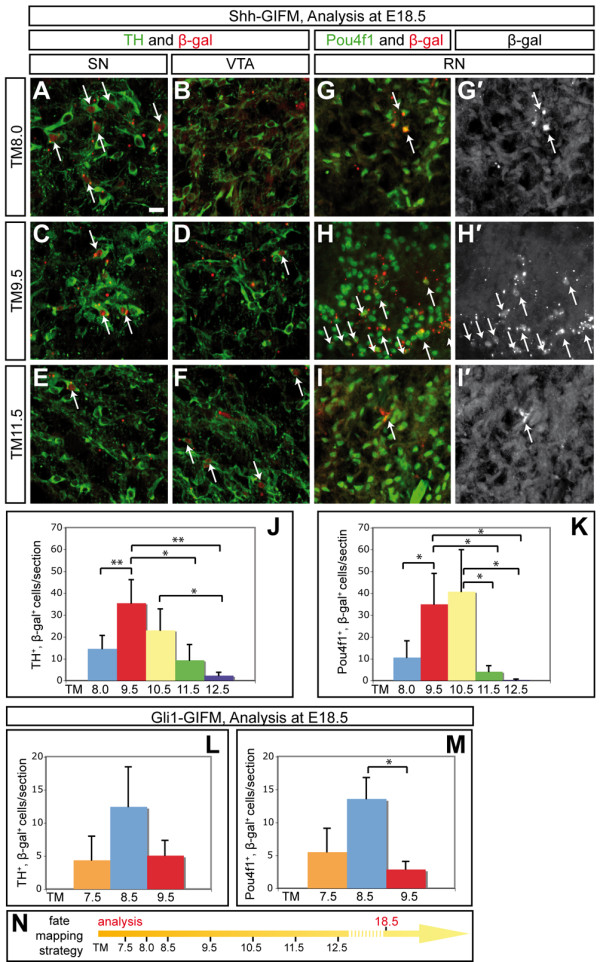
**Progenitors marked with Shh- and Gli1-GIFM give rise to DA and RN neurons over several days of embryonic development**. **(A-K) **Shh-GIFM; **(L,M) **Gli1-GIFM. (A-F) Immunofluorescent staining for DA neurons (TH, green) and β-gal-positive fate-mapped cells (red) on E18.5 coronal sections. Examples shown are located in the SN or the VTA. Arrows indicate double-labeled cells. Note that there are distinct contributions of marked cells to the SN or VTA at different fate-mapping time points. (G-I') Immunofluorescent staining for RN neurons (Pou4f1, green) and β-gal-positive fate-mapped cells (red) on E18.5 coronal sections. Arrows indicate double-labeled cells. Scale bar: 20 μm. (J-M) Quantification of the contribution of cells marked with Shh- or Gli1-GIFM to DA and RN neurons. Analysis was performed at E18.5. Cells positive for TH and β-gal (J,L) or Pou4f1 and β-gal (K,M) were counted and normalized for the number of counted sections. The peak contribution of cells marked with Shh-GIFM to DA neurons is with TM9.5, and to RN neurons with TM9.5 and TM10.5. These TM time points correlate with the broadest *Shh*-expressing domain. The peak contributions of cells marked with Gli1-GIFM to DA and RN neurons are one to two days earlier than observed for cells labeled with Shh-GIFM, consistent with medial *Gli1 *expression preceding *Shh *expression. Error bars indicate standard deviations; asterisks indicate the *P*-value as determined by Student's *t*-test (*P < 0.05; **P < 0.01). **(N) **Schematic of fate mapping strategy.

**Figure 5:**
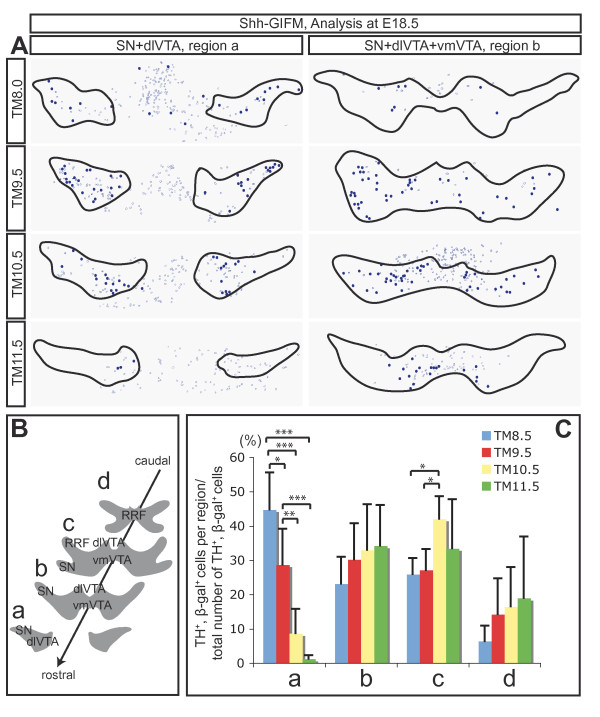
**Precursors labeled with Shh-GIFM at different time points contribute differentially to different rostral-caudal regions of DA neurons**. **(A) **Representative schematics of sections immunostained for TH and β-gal. β-gal- and TH-expressing cells (dark blue dots) and β-gal-expressing cells negative for TH (dark blue circles) on rostral (region a) and intermediate (region b) coronal sections through the E18.5 ventral midbrain. The DA neuron containing areas are outlined. Early (TM administration at E8.0 (TM8.0) to TM10.5) but not late Shh-GIFM (TM11.5 to TM12.5) results in contribution to the rostral and lateral areas of the SN. Shh-derived cells with TM11.5 contribute primarily to caudal and medial areas of the VTA. dlVTA, dorsal-lateral VTA; vmVTA, ventral-medial VTA. **(B) **Schematic of the regions used to quantify the contribution of fate-mapped cells to DA neurons in a region-specific manner. RRF, retrorubral field. **(C) **Relative contribution of Shh-derived cells to different regions of DA neurons along the rostral-caudal axis of the midbrain. For each animal (n ≥ 3), β-gal- and TH-co-expressing cells were counted in four regions along the rostral-caudal axis of the ventral midbrain as indicated in (B) and normalized for the combined number of double-labeled cells counted in the four regions (in percent). Error bars indicate standard deviation. Significance (*P < 0.05; **P < 0.01; ***P < 0.001) was determined by analysis of variance (ANOVA) and least significant difference (LSD) post-hoc analysis.

**Figure 6:**
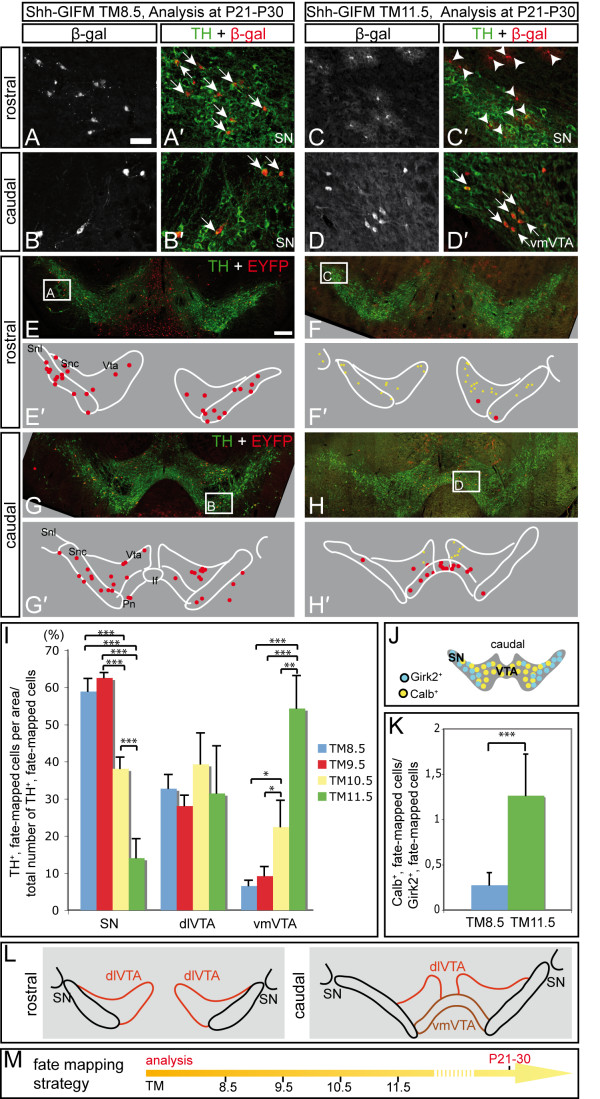
**Cells labeled with Shh-GIFM at different time points have a changing potential to contribute to subpopulations of DA neurons**. **(A-H) **Immunofluorescent staining for β-gal-positive fate-mapped cells (Shh-GIFM at E8.5 or E11.5) and DA neurons (TH) on coronal sections of the ventral midbrain (P21 to P30). The areas shown in (A-D) are indicated in (E-H). Arrows indicate double-labeled cells; arrowheads indicate β-gal-positive cells with astrocytic morphology. (E'-H') Representative schematics of the immunostained sections showing the distribution of TH-positive fate-mapped cells (red dots) and of fate-mapped cells with astrocytic morphology (yellow crosses). Rostral, Bregma -2.92; caudal, Bregma -3.40 [20]. If, interfascicular nucleus; Pn, paranigral nucleus; Snc, substantia nigra pars compacta; Snl, substantia nigra lateralis; Vta, VTA. Fate-mapped cells outside these areas are not represented. Cells with astrocytic morphology are not present with TM8.5. Scale bars: (A-D) 40 μm; (E-H) 200 μm. **(I) **Relative contribution of cells marked with Shh-GIFM between E8.5 and E11.5 to the SN (Snl + Snc), dorsal-lateral VTA (Vta) and ventral-medial VTA (Pn + If); see schematic in (L). For each animal (n ≥ 3), TH-positive fate-mapped cells were counted in the three indicated areas and normalized for the combined number of overlapping cells counted in these areas (in percent). Error bars indicate standard deviation. Significance (*P < 0.05; **P < 0.01; ***P < 0.001) was determined by ANOVA and LSD post-hoc analysis. **(J) **Distribution of Calbindin- and Girk2-positive cells. **(K) **Relative contribution of fate-mapped cells to Calbindin (VTA) versus Girk2 (SN) positive cells. Calbindin- or Girk2-positive fate-mapped cells were counted in three different rostral-caudal midbrain areas (n ≥ 3). The ratio of Calbindin-positive fate-mapped cells to Girk2-positive fate-mapped cells was determined. Significance (***P < 0.001) was determined by Student's *t*-test. **(L) **Schematic showing the SN, dlVTA and vmVTA. **(M) **Fate mapping strategy.

To investigate in a quantitative manner which neuronal subclasses develop from *Shh-*expressing precursors from different medial-lateral domains, we fate mapped *Shh*-expressing progenitors at different time points and analyzed the marked cells at E18.5 (Figure 4). Immunostaining for β-gal and TH showed that Shh-derived cells contributed to DA neurons with TM8.0 to TM12.5 (Figure 4A-F and data not shown). In contrast, with TM7.5, only a very small number of scattered cells expressed β-gal in the ventral midbrain, of which only rare cells were double-labeled with TH (data not shown). Quantitative analysis of the fate of progenitors marked at different time points demonstrated that the peak contribution of *Shh*-expressing cells to DA neurons was with TM9.5 (labeling at E10 to E10.5; Figure 4J). This peak contribution correlates with the time point of most extensive marking of *Shh*-expressing cells within the *Lmx1a*-positive putative DA precursor domain (Figure 2; Additional file 2). Next we analyzed whether the fate-mapped β-gal-positive cells expressed Pou4f1, a marker for neurons of the RN. We found contribution to the neurons of the RN with TM8.0 to TM11.5, but observed a high contribution to the RN only with TM9.5 and TM10.5 (Figure 4K). With TM8.0, the contribution to Pou4f1-positive RN neurons was largely restricted to the anterior RN (data not shown), consistent with our observation that at E10.5 only a few TM8.5 Shh-derived precursors expressed Nkx6-1 (Figure 2L,L'). The minor contribution of *Shh*-expressing progenitors to RN neurons after E11.5 is likely due to RN neurogenesis ceasing before recombination (marking) occurs in the ventricular zone [31]. We did not detect any overlap of fate-mapped cells with Islet1-expressing motoneurons of the oculomotor nucleus (data not shown), likely because motoneurons are born before *Shh *expression extends into the Nkx6-1-positive motoneuron precursor zone [31]. Furthermore, β-gal-positive neurons were never found in the area of the SN reticularis, which is located ventral to the SN, indicating that Shh-derived cells do not contribute to the GABAergic neurons of the SN reticularis (Figures 5A and 6E-H and data not shown).

In summary, we demonstrate that *Shh*-expressing mesencephalic progenitors give rise to DA neurons between E8.5 and E12.5 and to RN neurons between E8.5 and E10.5. The highest contribution to DA neurons occurs at the time point when *Shh *is expressed throughout the *Lmx1a*-positive DA precursor domain. We further observed that the highest contribution to RN neurons, which are likely derived from Nkx6-1 expressing precursors, occurs after the expansion of *Shh *expression into the Nkx6-1-positive domain.

### Progenitors expressing *Gli1 *between E7.5 and E9.5 give rise to dopaminergic and red nucleus neurons

**Figure 7:**
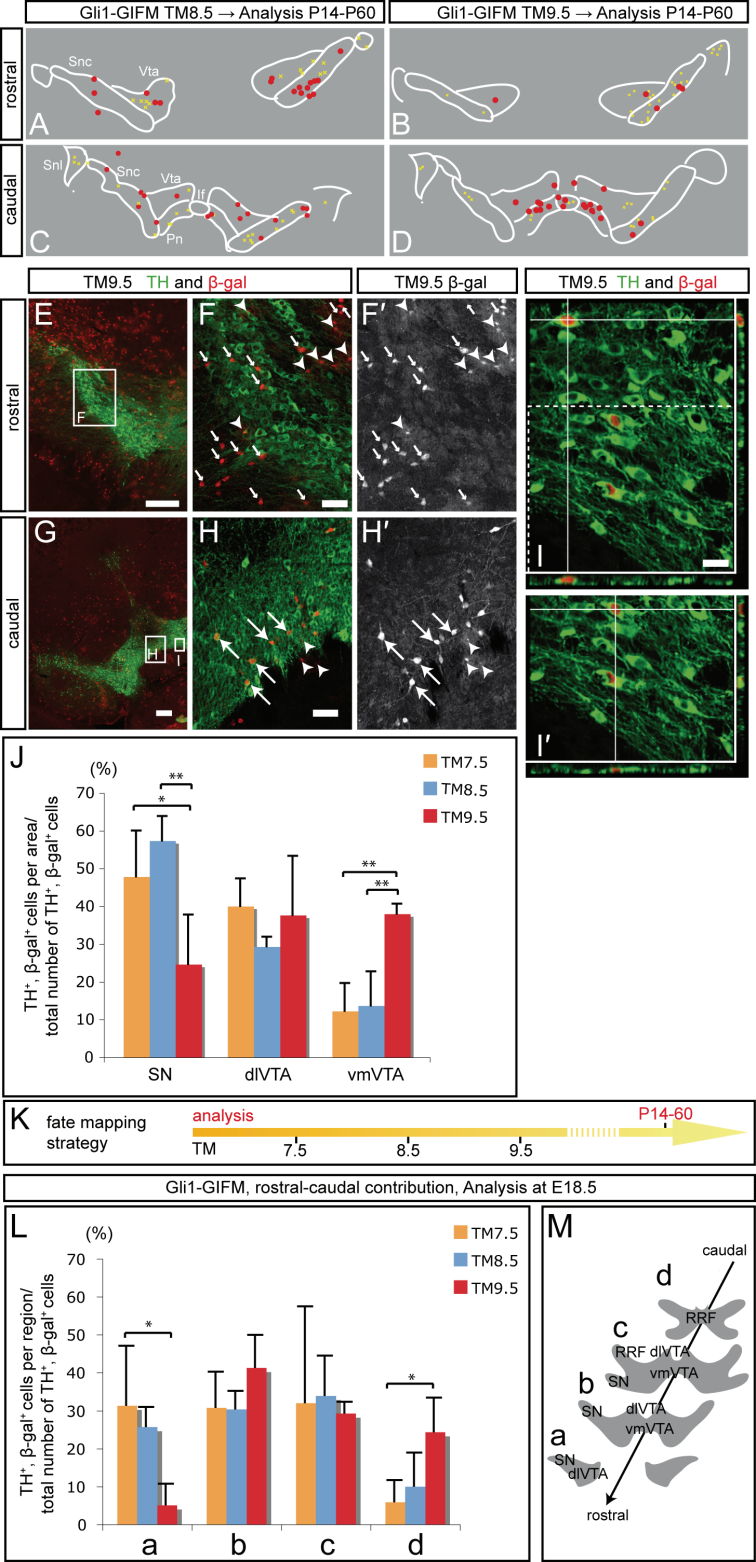
**Cells labeled with Gli1-GIFM at different time points have a changing potential to contribute to subpopulations of DA neurons**. **(A-D) **Representative schematics of immunostained sections (P14 to P60) labeled with Gli1-GIFM at E9.5. Red dots, TH-positive fate-mapped cells; yellow crosses, cells with astrocytic morphology. Rostral, Bregma -2.92; caudal, Bregma -3.40 [20]. If, interfascicular nucleus; Pn, paranigral nucleus; Snc, substantia nigra pars compacta; Snl, substantia nigra lateralis; Vta, VTA. **(E-I**'**) **Immunofluorescent staining for DA neurons (TH, green) and fate-mapped cells (Gli1-GIFM at E9.5; β-gal, red) on coronal midbrain sections. The areas in (F,F'), (H,H') and (I) are indicated in (E) and (G). Big arrows indicate TH- and β-gal-positive cells; arrowheads indicate fate-mapped cells with astrocytic morphology; small arrows indicate β-gal-positive, TH-negative cells with neuronal morphology. (I,I') Z-stacks of optical sections taken with a Zeiss Apotome. (I') Area indicated with dashed box in (I). Scale bars: (E,G) 200 μm; (F,H) 40 μm; (I,I') 20 μm. **(J) **Relative contribution of cells marked with Gli1-GIFM between E7.5 and E9.5 to the SN, dlVTA and vmVTA; see schematic in Figure 6L. For each animal (n ≥ 3), TH-positive fate-mapped cells were counted in the three indicated areas and normalized for the combined number of overlapping cells counted in these areas (in percent). Error bars indicate standard deviation. Significance (*P < 0.05; **P < 0.01) was determined by ANOVA and LSD post-hoc analysis. **(K) **Fate mapping strategy. **(L) **Relative contribution of fate-mapped cells to different rostral-caudal midbrain regions at E18.5. For each animal (n ≥ 3), β-gal- and TH-co-expressing cells were counted in four rostral-caudal midbrain regions (see (M)) and normalized for the combined number of cells counted in the four regions (in percent). Error bars indicate standard deviation. Significance (*P < 0.05; **P < 0.01) was determined by ANOVA and LSD post-hoc analysis. **(M) **Rostral-caudal areas used to quantify the contribution of fate-mapped cells to DA neurons at E18.5. RRF, retrorubral field.

To investigate the contribution of Shh-responding ventral mesencephalic progenitors to different midbrain neurons, we marked these domains at earlier time points using Gli1-GIFM. *Gli1*-expressing progenitors gave rise to DA and RN neurons when marked with TM at E7.5, E8.5 and E9.5, with the highest contribution observed with TM8.5 (Figure 4L,M). We observed, however, a much lower number of fate-mapped cells that overlapped with DA or RN neurons than with Shh-GIFM, consistent with the low recombination efficiency of the *Gli1*^*CreER *^allele observed in the ventral midbrain precursor domain (Figures 2 and 3; Additional file 2). In addition to RN and DA neurons, motoneurons of the oculomotor nucleus were derived from *Gli1*-expressing precursors with TM8.5 (data not shown), consistent with *Gli1 *being expressed in the Nkx6-1-positive motoneuron precursor zone before motoneuron differentiation [31]. Marking with TM9.5 and TM10.5 resulted in the labeling of TH-negative cells within the SN and cells in the SN reticularis (Figure 7E-G and data not shown). This is in accordance with the observation that Gli1-GIFM at E9.5 and E10.5 results in the marking of Gli1-derived precursors in the Nkx2-2-positive precursor domain (Figure 2V,X and data not shown). In conclusion, the contribution of Gli1-derived cells to DA (and RN neurons) was earlier than that of Shh-derived cells (Gli1-GIFM, TM7.5 to TM9.5; Shh-GIFM, TM8.0 to TM11.5), consistent with the dynamic and sequential *Shh *and *Gli1 *gene expression patterns in the ventral mesencephalon. Furthermore, Gli1-derived cells contributed to a wider array of ventral midbrain cell types, consistent with the earlier and broader expression of *Gli1 *in the ventral midbrain precursor domain.

### The potential of *Shh-*expressing progenitors to contribute to different groups of dopaminergic neurons changes over time

Since DA neurons of the ventral midbrain are organized into several anatomically and functionally distinct subclasses [1], we next asked whether a differential temporal-spatial origin of DA neurons might correspond to a preferential localization in specific DA nuclei. We first determined the fate of ventral midbrain progenitors using Shh-GIFM, since the recombination efficiency was higher and the medial-lateral domains at the initial time points were more compact than with the Gli1-GIFM approach. We divided the ventral midbrain into four rostral-caudal regions, based on the presence of different DA nuclei (Figure 5B). The most rostral region contains SN and dlVTA neurons (region a), at slightly more caudal levels DA neurons are distributed into SN, dlVTA and vmVTA (region b), further caudally the retrorubral field becomes visible (region c), and in the most caudal sections DA neurons are organized into the retrorubral field (region d). Figure 5A schematically shows representative sections of marked cells in rostral and intermediate regions (region a and b) at E18.5 for each time point of TM administration. With TM8.0, Shh-derived cells were mainly found in rostral regions whereas with TM9.5 cells contributed to the entire rostral-caudal and medial-lateral extent of the DA nuclei. With TM10.5, the contribution shifted more caudally, and with TM11.5, cells were distributed primarily in caudal and medial regions.

To quantitatively assess the distribution of fate-mapped cells marked at the different time points, β-gal and TH double-labeled cells were counted in each region and the relative and absolute contribution to each region was determined (Figure 5C; Additional file 4A). The relative contribution is a measure for the contribution of fate-mapped/TH-positive cells to a region or area in relation to the total number of fate-mapped/TH-positive cells counted (Figures 5C, 6I and 7J,L; see Materials and methods for details). Since GIFM results in random mosaic labeling of a subset of cells expressing a particular gene and the recombination efficiency can vary between animals and time points, this approach provides an unbiased assessment of the contribution of cells marked at different time points to different DA nuclei. Comparing the fate of cells marked between E8.0 and E11.5, the quantitative analysis confirmed that there was a continuous decrease in the relative contribution of Shh-derived cells to the most rostral region (region a). In contrast, there was a slight increase in the relative contribution of Shh-derived cells to more caudal regions (region c) when comparing TM10.5 with TM8.0 or TM9.5 (Figure 5C and data not shown). This shift from rostral to caudal was also evident when comparing the actual number of Shh-derived cells giving rise to DA neurons. Comparison of cells labeled with TM9.5 or TM10.5 (the two time points with the highest number of labeled cells), showed no significant changes in the number of cells contributing to caudal regions (regions b to d), but a significantly lower contribution of cells labeled with TM10.5 to the most rostral region (region a; Additional file 4A). These data indicate that the contribution of Shh-derived cells to DA neurons shifts from rostral to caudal when Shh-derived cells are marked at progressively later stages.

To further investigate the changing potential of Shh-derived cells to contribute to different midbrain DA subgroups, we analyzed the distribution of cells labeled between E8.5 and E11.5 in postnatal brains (P21 to P30) because the distinct anatomy of the different DA subgroups is more obvious at mature stages (Figure 6E-H,L). We focused our analysis on the SN and VTA because the subdivisions of the SN and VTA can be clearly determined based on their location: the SN is divided into the SN lateralis and SN pars compacta. The VTA region is subdivided into the dorsal-lateral parabrachial pigmented nucleus (referred to as dlVTA) and the ventral-medial paranigral nucleus and interfascicular nucleus (referred to as vmVTA; Figure 6E',G',L) [20]. To assess the distribution of DA neurons within these three areas, we counted all TH-positive neurons on sections representative of different rostral-caudal levels of the ventral midbrain and determined the relative number of TH-positive neurons in SN, dlVTA and vmVTA. We found that 52 .0 ± 2.0% of the DA neurons were located in the SN, 33.2 ± 1.6% in the dlVTA and 14.8 ± 0.4% in the vmVTA (n = 3; Additional file 4E,F). We next performed a quantitative analysis of the relative contribution (the contribution of fate-mapped/TH-positive cells to an area in relation to the total number of fate-mapped/TH-positive cells counted) of Shh-derived cells to DA neurons in the SN, dlVTA and vmVTA (see Materials and methods for details). This analysis demonstrated that with TM8.5 and TM9.5, about 60% of the TH-positive Shh-derived neurons were located in the SN (59.0 ± 3.4% and 62.6 ± 1.3%, respectively), about 30% in the dlVTA (32.8 ± 3.7% and 28.1 ± 2.8%, respectively), and about 10% of Shh-derived DA neurons were located in the vmVTA (6.6 ± 1.5% and 9.3 ± 2.4%, respectively) (Figure 6I). Comparison of the relative contributions of TH-positive neurons fate-mapped with TM8.5 and TM9.5 and the distribution of DA neurons to these three areas indicates that Shh-expressing cells labeled with TM8.5 and TM9.5 have a slight bias to contribute to the SN, but are underrepresented in the vmVTA (Figure 6I; Additional file 4E). This biased contribution is a rather surprising result for Shh-GIFM at E9.5, since at this labeling time point the *Lmx1a*-positive DA precursor domain is labeled extensively (Figures 2N,O and Figure 3C,D). Interestingly, with TM10.5 and TM11.5, the Shh-derived precursors had a markedly reduced potential to contribute to the SN (38.1 ± 3.1% and 14.1 ± 5.1%, respectively) and an increased potential to contribute to the vmVTA (22.5 ± 7.1% and 54.3 ± 8.7%, respectively) when compared to cells fate-mapped with TM8.5 or TM9.5 or when compared to the distribution of DA neurons (Figure 6I; Additional file 4F).

To further test whether the increase in the relative contribution to the vmVTA with TM11.5 is accompanied by a decreased contribution to the SN, we quantified the number of fate-mapped cells that gave rise to cells expressing Calbindin and Girk2. Calbindin is a calcium binding protein primarily expressed in the VTA. Girk2 is primarily expressed in the SN, but some positive cells are also found in the dlVTA (Figure 6J). When we compared the ratio of cells contributing to Calbindin- versus Girk2-positive cells, we observed that whereas with TM8.5 more cells contributed to Girk2 than to Calbindin-positive cells, the opposite was the case with TM11.5 (Figure 6K).

To investigate whether the shift in competence of precursors expressing *Shh *after E10.5 (TM10.5 and TM11.5) is due to an absolute decrease in cells contributing to the SN and/or an absolute increase in the number of cells contributing to the vmVTA, we also compared the actual numbers of Shh-derived cells contributing to the SN, dlVTA and vmVTA. This analysis revealed that, compared to Shh-derived cells labeled with TM9.5 (the peak contribution to DA neurons), there was a significant decrease in the number of Shh-derived cells giving rise to the SN and dlVTA with TM11.5 (and with TM10.5 for the SN). This result is not surprising when taking into account that the overall contribution of Shh-derived cells to DA neurons is much lower with TM11.5 than with TM9.5. Importantly, however, the number of cells contributing to the vmVTA did not change significantly when comparing cells labeled with TM 9.5, TM10.5 and TM11.5 (Additional file 4C). In conclusion, these data indicate that ventral midbrain precursors, which are located lateral to the ventral midline and are labeled with Shh-GIFM after E10.5, have a reduced potential to generate DA neurons of the SN, but maintain the ability to give rise to the vmVTA. In contrast, medial progenitors labeled with Shh-GIFM at E8.5 and E9.5 can contribute to all DA neuronal subpopulations.

### The distinct developmental potential of precursors is not solely due to different birthdates of dopaminergic neuron subclasses

DA neurons are born between E10.5 and E14.5 in the mouse according to [3H] thymidine birthdating studies [34]. vmVTA neurons (interfascicular, paranigral) appear to be born a day later (peak at E12.5) than SN or dlVTA neurons (peak at E11.5). Moreover, rostral DA neurons (peak at E11.5) are born before caudal DA neurons (peak at E12.5) [34]. Therefore, the significant decrease in the potential of Shh-derived cells to give rise to the SN with TM10.5 and TM11.5 labeling (marking at E11 to E13) compared to cells labeled with TM8.5 and TM9.5 might be due to the earlier birth date of SN neurons. If most SN and dlVTA neurons are born before E12.0, TM administration at E11.5 will selectively label progenitors that give rise to the vmVTA, whereas TM administration at E8.5 and E9.5 should label early and late born DA neurons that give rise to all subpopulations. Alternatively, the medial and lateral putative DA precursor domains (domains 1 and 2, Figure 2Z) could be intrinsically different, as supported by the molecularly distinct medial domain that expresses *Corin *and *Msx1 *and ceases to respond to Shh earlier than the lateral DA precursors. To investigate whether the medial-lateral location of precursors might also contribute to different progenitor fates, we took advantage of Gli1-GIFM, since TM administration at E9.5 (labeling between E10 and E11) marked Gli1-derived progenitors in a lateral domain that included the posterior-lateral *Lmx1a*-positive domain (Figures 2X,Y and 3M,N; Additional file 2H). Since the labeling occurs a day before the majority of SN and VTA neurons are born [34], a preferential contribution of lateral *Gli1*-expressing progenitors to the vmVTA would indicate that they are intrinsically different from the other DA precursors. Indeed, analysis of postnatal brains (P14 to P60) showed that with TM9.5, the relative contribution of Gli1-derived cells to the SN was significantly reduced compared to the normal distribution of DA neurons or compared to cells fate-mapped with TM7.5 or TM8.5, whereas the relative contribution to the vmVTA was significantly increased with TM9.5 (Figure 7A-J and data not shown). When we compared the actual numbers of Gli1-derived cells contributing to the SN, dlVTA and vmVTA at each stage, we also found that there was an increase in the number of Gli1-derived cells giving rise to the vmVTA with TM9.5 compared to TM7.5 and TM8.5, and a significant decrease in the number of cells contributing to the SN with TM9.5 compared to TM8.5 (Additional file 4D). In conclusion, these data provide strong evidence that precursors, which are located lateral to the ventral midline and continue to respond to Shh signaling after E9.5 (Gli1-GIFM at E9.5), preferentially contribute to the vmVTA.

Finally, we determined whether, similar to the change in rostral-caudal distribution at E18.5 of cells marked with Shh-GIFM, the rostral-caudal distribution of cells labeled with Gli1-GIFM changed with different fate-mapping time points. To this end, β-gal and TH double-labeled cells were counted in four rostral-caudal regions at E18.5 and the contribution to each region was determined (Figure 7 L,M; Additional file 4B). As expected, there was a decrease in the relative contribution of Gli1-derived cells to the most rostral region with TM9.5 compared to TM7.5. In contrast, there was a continuous increase in the relative contribution of *Gli1*-expressing precursors to the most caudal region (region d) with TM7.5 to TM9.5 (Figure 7L). This shift from rostral to caudal contribution was also evident when the actual number of Gli1-derived cells giving rise to DA neurons was compared at E18.5 (Additional file 4B). These data indicate that the contribution of Gli1-GIFM cells to DA neurons shifts from rostral to caudal when Gli1-derived cells are marked at progressively later stages.

### *Shh- *and *Gli1*-expressing lateral progenitors give rise to astrocytes

**Figure 8:**
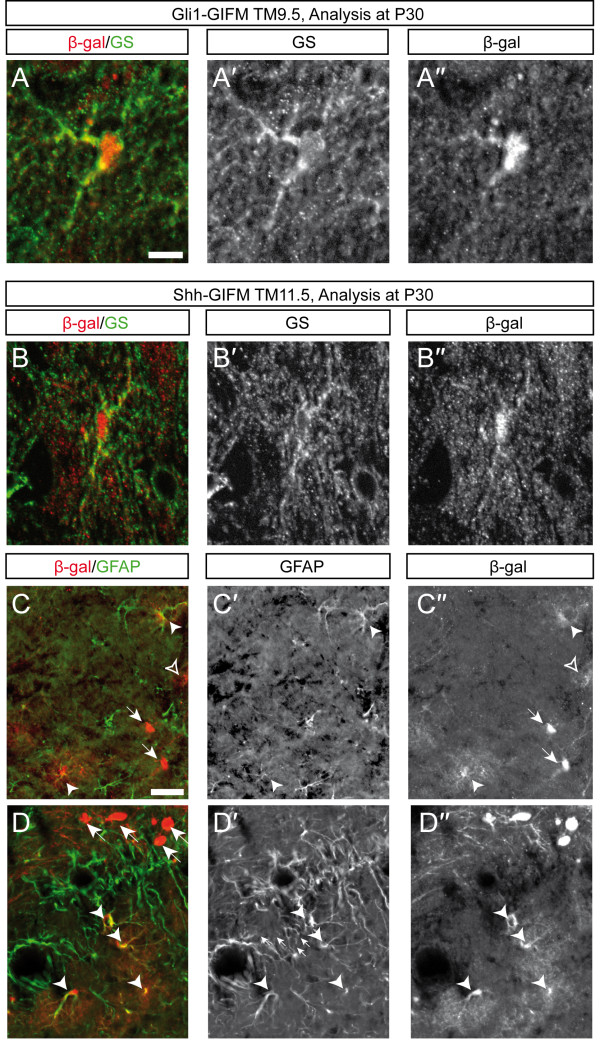
**Fate-mapped cells contribute to midbrain astrocytes**. **(A-D) **Immunostaining for glutamine synthetase (GS) and β-gal (A,B) or GFAP and β-gal (C,D). Sections were imaged using a Zeiss Apotome setup. (A,B) β-gal-positive cells with astrocytic morphology overlap with GS. (C,D) Note that not all β-gal-positive cells with astrocytic morphology express GFAP. Filled arrowheads indicate cells that co-express GFAP; open arrowheads indicate cells that do not express GFAP; arrows highlight cells with a neuronal morphology. Scale bars: (A-B'') 10 μm; (C-D'') 40 μm.

In addition to uncovering the progressive temporal change in the distribution of Shh-derived cells to DA nuclei, we found that with Shh-GIFM at E9.5 to E11.5, fate-mapped cells in the DA neuron containing areas had distinct morphologies. In addition to neurons with large, brightly labeled cell bodies and axonal projections and/or dendrites, cells with small cell bodies and a 'halo' of weakly labeled, small and highly branched processes were detected within the SN and VTA areas (TM9.5, 19 of 21 sections; TM11.5, 19 of 19 sections; Figure 6C,F,H and data not shown). Since the latter morphology is indicative of astrocytes, we performed immunostainings for β-gal and glutamine synthetase, an established broad astrocyte marker [35,36] or for β-gal and the astrocyte marker GFAP [37]. We found that β-gal-positive cells with an astrocytic morphology indeed expressed glutamine synthetase and GFAP (Figure 8B-D). Consistent with the Shh-GIFM results, Gli1-derived cells labeled with TM8.5 and TM9.5 also gave rise to cells with astrocytic morphology (Figures 7D,F,H and 8A and data not shown). Notably, cells with astrocytic morphologies were less often observed in the DA neuron-containing areas with Shh GIFM TM8.5 (astrocytes in 8 of 40 sections; Figure 6A,B,E,G and data not shown) or Gli1 GIFM TM7.5 (24 of 39 sections; data not shown), indicating that the medial precursor population marked with these GIFM schemes has a low gliogenic potential.

## Discussion

By determining the fate of ventral midbrain cells arising from progenitors that express either *Shh *or *Gli1 *at different developmental time points, we were able to establish a detailed quantitative assessment of the contribution of distinct ventral progenitor domains to different DA nuclei, RN neurons and astrocytes. We show that progenitors marked with Shh-GIFM at E8.0 to E11.5 and with Gli1-GIFM at E7.5 to E9.5 give rise to DA neurons. We further uncovered that lateral precursors that are labeled with Shh-GIFM after E10.5 (TM10.5 and TM11.5) preferentially give rise to DA neurons in the vmVTA. In light of birth-dating studies, the shift in potential could be solely linked to a later birthdate of vmVTA than SN DA neurons [34]. However, when we traced the fate of *Gli1*-expressing progenitors, which are located lateral to the ventral midline at earlier stages (TM9.5 Gli1-GIFM), we found that these cells also had an increased potential to contribute to DA neurons in the vmVTA. These results suggest that the distinct developmental fate of the lateral progenitors that we observed with Shh-GIFM at E11.5 and Gli1-GIFM at E9.5 is due, at least in part, to an intrinsic difference between medial and lateral progenitors in the ventral midbrain. Indeed, factors such as Corin and Msx1 appear to be more medially restricted within the broad Lmx1a expression domain, which is thought to give rise to all DA neurons (Figure 2A-H) [23,30]. Alternatively, our data could support a model in which DA neurons in the vmVTA do not arise from the Lmx1a-positive domain, but rather arise from the more lateral Nkx6-1 domain. This idea is based on the observations that only very few precursor cells are labeled within the Lmx1a-positive domain with Gli1-GIFM at E9.5, but we still find substantial contribution of the fate-mapped cells to the vmVTA. Finally, *Shh*- and *Gli1-*expressing precursors that overlap with the lateral Lmx1a, Nkx6-1 and Nkx2-2 domains also give rise to ventral midbrain astrocytes and RN neurons, but the most medially located precursors that are labeled with Shh-GIFM at E8.5 or Gli1-GIFM at E9.5 are much less gliogenic and produce only few RN neurons. The ventral progenitor region of the mesencephalon thus has a higher degree of complexity than was previously appreciated.

A recent GIFM study using *Shh*^*CreERneo *^concluded that progenitors from distinct medial-lateral domains in the ventral mesencephalon give rise to DA and RN neurons [9]. It was also proposed that different ventral *Shh*-expressing progenitor domains might give rise to distinct DA neurons [9]. In that study, three precursor domains were delineated based on the location of fate-mapped cells in the E10.5 to E13.5 mesencephalon: a medial (TM7.5), a medial-lateral (TM8.0) and a lateral (TM11.5 and TM12.5) domain. With TM7.5 Shh-GIFM, that study observed β-gal-positive cells in the vmVTA and in the overlying rostral linear nucleus at E18.5. However, immunostaining for TH and β-gal showed that very few fate-mapped cells were DA neurons. Largely based on the distribution of β-gal-positive (X-gal-labeled) cells in E18.5 midbrain sections, the authors further concluded that Shh-GIFM with TM8.5 resulted in labeling of all subsets of DA neurons, whereas precursors labeled with TM11.5 or TM12.5 contributed to the RN and the vmVTA. However, the study did not identify which neuronal subtypes expressed β-gal in the adult, and did not distinguish between marked neurons and glia for most labeling time points. Since we found that Shh-derived astrocytes are intermingled with DA and RN neurons, the distinction of different cell types is a prerequisite for making qualitative and quantitative conclusions about the contribution of fate-mapped cells to different ventral midbrain cell types. Indeed, we also find that medial cells are labeled in the anterior midbrain with TM7.5, but only rarely do these cells express TH (data not shown). Similarly, with TM11.5 we observed cells in the area of the RN, but marker and morphological analysis revealed that few fate-mapped cells expressed Pou4f1 and most had an astrocytic morphology (Figures 4I,K and 6F,H and data not shown).

In addition to delineating different precursor pools, the dissection of the fate of Shh-responding (*Gli1*-expressing) and *Shh*-expressing cells provides new insight into when Shh signaling might be required for induction of DA and RN neurons. *Gli1 *expression is a well-established readout for Shh signaling in the developing embryo, as *Gli1 *expression is dependent on activator functions of Gli2 and Gli3 [10,11]. While it cannot be fully excluded that *Gli1 *transcription might also be activated by other signaling pathways in some tissues or tumors [38], we have previously shown that inactivation of Shh signaling in the midbrain between E8.5 and E9.0 results in the rapid and complete loss of *Gli1 *expression [13]. Based on the changing expression patterns of *Shh *and *Gli1 *and our GIFM results, we propose the following timeline for Shh signaling in the ventral midbrain (compare Figure 2Z): a medial, largely Lmx1a-positive progenitor domain gives rise predominantly to DA neurons and receives only a brief high level of Shh signaling from the underlying notochord around E8.0 (Gli1-GIFM at E7.5, domain 1). The progenitors in this medial domain subsequently cease responding to Shh (and stop expressing *Gli1*) once they start expressing *Shh *(Shh-GIFM at E8.5 and Gli1-GIFM at E9.5). Progenitors in the adjacent domain that spans the lateral Lmx1a domain, the Nkx6-1 domain and the medial part of the Nkx2-2 domain receive a high level of Shh signaling when *Shh *is induced in cells of the medial floor plate after E8.5 (Gli1-GIFM at E8.5). Most of these progenitors cease responding to Shh after E9.5 and switch on *Shh *expression (Gli1-GIFM at E9.5 and Shh-GIFM at E9.5). Progenitors in the ventral-lateral domain thus receive Shh signaling after E8.5 and they give rise to DA neurons, RN neurons, oculomotor neurons and astrocytes. Finally, progenitors in a more lateral, Nkx2-2-positive domain only respond strongly to Shh signaling after the lateral expansion of the Shh expression domain (Gli1-GIFM at E9.5 and Shh-GIFM at E9.5) and give rise to neurons in the SN reticularis. Surprisingly, some cells in the Lmx1a and Nkx6-1-positive domain in the posterior midbrain sustain their ability to respond to Shh signaling after E9.5, indicating that the switch from Shh-responsiveness to *Shh*-expression might not be an all-or-nothing event in some cells, or occurs in a progressive manner from anterior to posterior. A gradual switch is supported by the RNA *in situ *hybridization data, which show that, at E9.5, weak/mosaic *Shh *expression at the lateral edge of the *Shh *domain partially overlaps with weak/mosaic *Gli1 *expression at the medial edge of the *Gli1 *domain (Figure 1B,G). Interestingly, even though *Shh *is eventually downregulated medially, medial progenitors do not initiate a second high-level response to Shh signaling, as judged by the absence of medial *Gli1 *expression at E11.5 and E12.5 (Figure 1). The temporally dynamic expression and signaling of Shh in the mesencephalon we describe here is distinct from the classical morphogen model of Shh in the spinal cord, where neuronal progenitor cells are continuously exposed to Shh and interpret the Shh signal based on the concentration and duration of Shh signaling [39]. The model is, however, consistent with the recently proposed mechanisms for the induction of the floor plate in the spinal cord, which is based on a changing response of floor plate precursors to Shh signaling. Initially, the induction of spinal cord floor plate identity requires high levels of Shh signaling from the underlying notochord. Subsequently, in order to establish full floor plate identity, the precursors must cease responding to Shh signaling by extinguishing *Gli2 *and *Gli3 *expression [40].

Is our proposed model of Shh signaling supported by studies of loss-of-function in components of the Shh pathway? Complete inactivation of Shh signaling in *Shh *null mutants results in the loss of all ventral midbrain structures [13,41]. Thus, Shh signaling is essential for DA, RN and motoneuron induction. Using conditional knock-out analysis to inactivate Shh signaling by deleting the Shh receptor *Smoothened *(*Smo*) in the mid/hindbrain at different time points, we previously showed that some DA neurons only require Shh signaling before E9.0, since a small number of DA neurons are induced when Shh signaling is inactivated at E9.0. In contrast, inactivation of Shh signaling at E11.0 has no effect on the specification of DA neurons [13]. It was further demonstrated that Islet1-positive motoneurons are completely lost when *Smo *or *Shh *are inactivated at E9.0 in the mid/hindbrain [13,42]. Consistent with these previous findings, our present fate-mapping data show that Shh-responding (*Gli1*-expressing) progenitors contribute to DA neurons only before E10.5 (TM7.5 to TM9.5) and to oculomotor neurons after E8.5 (TM8.5).

Given the finding that progenitors lateral to the ventral midline that are labeled with Shh-GIFM at E11.5 or Gli1-GIFM at E9.5 preferentially give rise to vmVTA DA neurons and therefore have a more restricted fate potential than progenitors in the ventral midline, it is tempting to speculate that intrinsic molecular differences in spatially segregated DA progenitor populations are causally linked to the distinct physiological and functional properties of the DA neurons they give rise to. For example, DA neurons that project to corticolimbic targets are primarily located in the vmVTA and have unconventional fast-firing properties and small dopamine transporter/TH mRNA expression ratios. In contrast, SN DA neurons and dlVTA DA neurons project to the dorso-lateral striatum and the lateral shell of the nucleus accumbens, respectively, and have slow-firing properties and a high dopamine transporter/TH mRNA expression ratio [43].

## Conclusions

Our study provides a spatial-temporal genetic fate map of the ventral mesencephalic precursors based on the dynamic expression of *Shh *and *Gli1*. Since *Gli1 *expression is a readout for Shh signaling, our GIFM analysis also gives insight into when and where different ventral midbrain progenitors receive high levels of Shh signaling and suggests that the timing and duration of Shh signaling might influence the type of DA neuron a particular progenitor becomes. In conclusion, our study establishes a direct link between the location, distinct gene expression and Shh-responsiveness of ventral midbrain precursors and the fate of their descendants in the adult murine midbrain.

## Abbreviations

ANOVA: analysis of variance; β-gal: β-galactosidase; DA: dopaminergic; dlVTA: dorsal-lateral VTA; E: embryonic day; EYFP: enhanced yellow fluorescent protein; GFAP: glial fibrillary acidic protein; GFP: green fluorescent protein; GIFM: genetic inducible fate mapping; LSD: least significant difference; P: postnatal day; RN: red nucleus; Shh, Sonic Hedgehog; SN: substantia nigra; TH: tyrosine hydroxylase; TM: tamoxifen; vmVTA: ventral-medial VTA; VTA: ventral tegmental area.

## Competing interests

The authors declare that they have no competing interests.

## Authors' contributions

SB and ALJ designed experiments and wrote the manuscript. SB, GOB, AK, SC, EM, AD and DS performed experiments. All authors read and approved the final manuscript.

## Supplementary Material

Additional file 1 **Comparison of the extent of labeling using the *Shh***^***CreERneo***^**and *Shh***^***CreER***^**alleles with *R26***^***lz***^**reporter mice**. Coronal sections of E18.5 brains were labeled with X-gal staining and counterstained with Fast Red. TM (4 mg) was given at E10.5. **(A-F) **Note that only few cells are labeled in the midbrain of *Shh*^*CreERneo*^^/^^*+*^*R26*^*lz*^^/^^*+ *^mice (A-C), while many cells are labeled in the midbrain of *Shh*^*CreER*^^/^^*+*^*R26*^*lz*^^/^^*+ *^mice (D-F). Pictures in the upper right corner are higher magnifications of the area indicated in the black box. Several images were taken for each area shown and stitched together using the Zeiss Mosaix software. Click here for file

Additional file 2 **Initial domains of cells marked with Shh- or Gli1-GIFM in comparison with Lmx1a**. **(A-H**'**) **TM was administered at the indicated time points and marked cells were analyzed at E12.5 on anterior and posterior coronal midbrain sections with EYFP (red) and Lmx1a (green) immunostaining. Note that on anterior sections there is no or only little overlap of Lmx1a with cells fate-mapped with Shh-GIFM at E11.5 or Gli1-GIFM at E9.5. Scale bars: (A-H) 100 μm; (B',D',F',H') 50 μm. **(I-L) **Number of fate-mapped cells overlapping with Lmx1a (I,K) or TH (J,L). Cells were counted on one anterior (ant), one intermediate (int) and one posterior (pos) coronal section for each E12.5 embryo (or at E11.5 for Shh-GIFM TM8.5). For one time point and one section level, each data point represents cell numbers from one embryo. Note that overlap of fate-mapped cells is much higher with Lmx1a than with TH, since Lmx1a is expressed in DA precursors and differentiated DA neurons and differentiation of DA neurons is not complete by E12.5. The trends observed at E12.5 correlate with the results of the analysis at E18.5 and in the adult brain sections: the highest contribution to DA neurons is observed with Shh-GIFM at E9.5 (I,J); cells marked with Shh-GIFM TM8.5 contribute preferentially to anterior DA neurons (J); cells marked with Shh-GIFM TM11.5 contribute preferentially to intermediate and posterior DA neurons (J); Gli1-GIFM results in less labeling than Shh-GIFM (compare (I,J) with (K,L)). Click here for file

Additional file 3 **Markers used to determine the location of Shh- and Gli1-derived precursors in relation to known ventral midbrain expression domains**. Click here for file

Additional file 4 **Contribution of fate-mapped cells to different regions of DA neurons along the rostral-caudal axis of the prenatal midbrain and to different subsets of DA neurons in the adult midbrain. (A,B) **Number of Shh-derived (A) or Gli1-derived (B) cells contributing to different rostral-caudal regions of DA neurons at E18.5. For each animal (n ≥ 3), β-gal- and TH-co-expressing cells were counted in four regions along the rostral-caudal axis of the ventral midbrain as indicated in Figure 5B and normalized for the number of sections counted for each region. **(C,D) **Number of Shh-derived (C) or Gli1-derived (D) cells contributing to different subsets of DA neurons in the adult brain. For each animal (n ≥ 3), β-gal- and TH-co-expressing cells were counted in the SN, dlVTA and vmVTA as indicated in Figure 6L and normalized for the number of sections counted for each region. **(E,F) **Relative distribution of DA neurons in the SN, dlVTA and vmVTA (black bars) compared to the relative contribution of Shh-GIFM marked cells to the three areas. For a clearer representation, the data were split into two diagrams. The data for the fate-mapped cells (TM8.5, TM9.5, TM10.5, TM11.5) are also shown in Figure 6I. Error bars indicate standard deviation. Significance (*P < 0.05; **P < 0.01; ***P < 0.001) was determined by ANOVA and LSD post-hoc analysis (A-D) or Student's *t*-test (E,F). Click here for file
