# Supplementary figures and images for: Temporal-spatial changes in Sonic Hedgehog expression and signaling reveal different potentials of ventral mesencephalic progenitors to populate distinct ventral midbrain nuclei
Source: Neural Dev. 2011 Jun 20;6:29. doi: 10.1186/1749-8104-6-29 (PMC3135491; doi:10.1186/1749-8104-6-29)

***ShhCreERneo-GIFM***

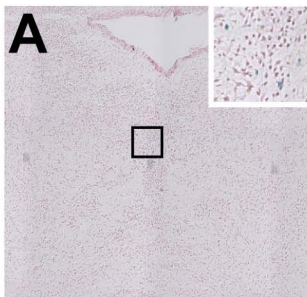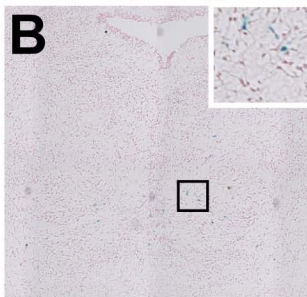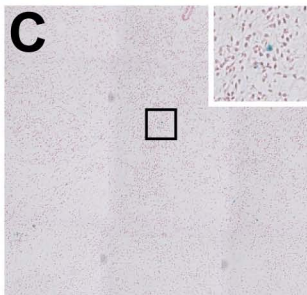

***ShhCreER-GIFM***

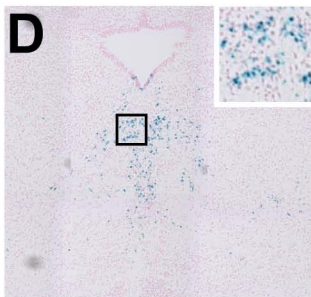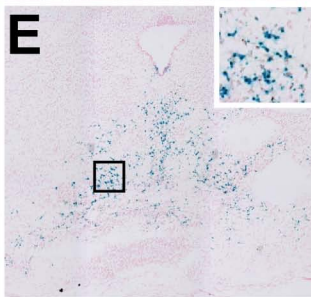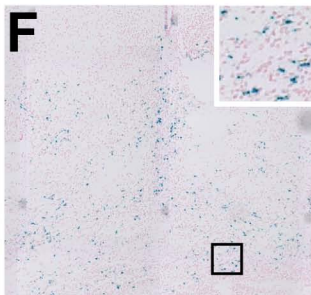

posterior

anterior

Supplement: Additional file 1 — Comparison of the extent of labeling using the ShhCreERneoand ShhCreERalleles with R26lzreporter mice. Coronal sections of E18.5 brains were labeled with X-gal staining and counterstained with Fast Red. TM (4 mg) was given at E10.5. (A-F) Note that only few cells are labeled in the midbrain of ShhCreERneo/+R26lz/+ mice (A-C), while many cells are labeled in the midbrain of ShhCreER/+R26lz/+ mice (D-F). Pictures in the upper right corner are higher magnifications of the area indicated in the black box. Several images were taken for each area shown and stitched together using the Zeiss Mosaix software. [file 1749-8104-6-29-S1.PDF]

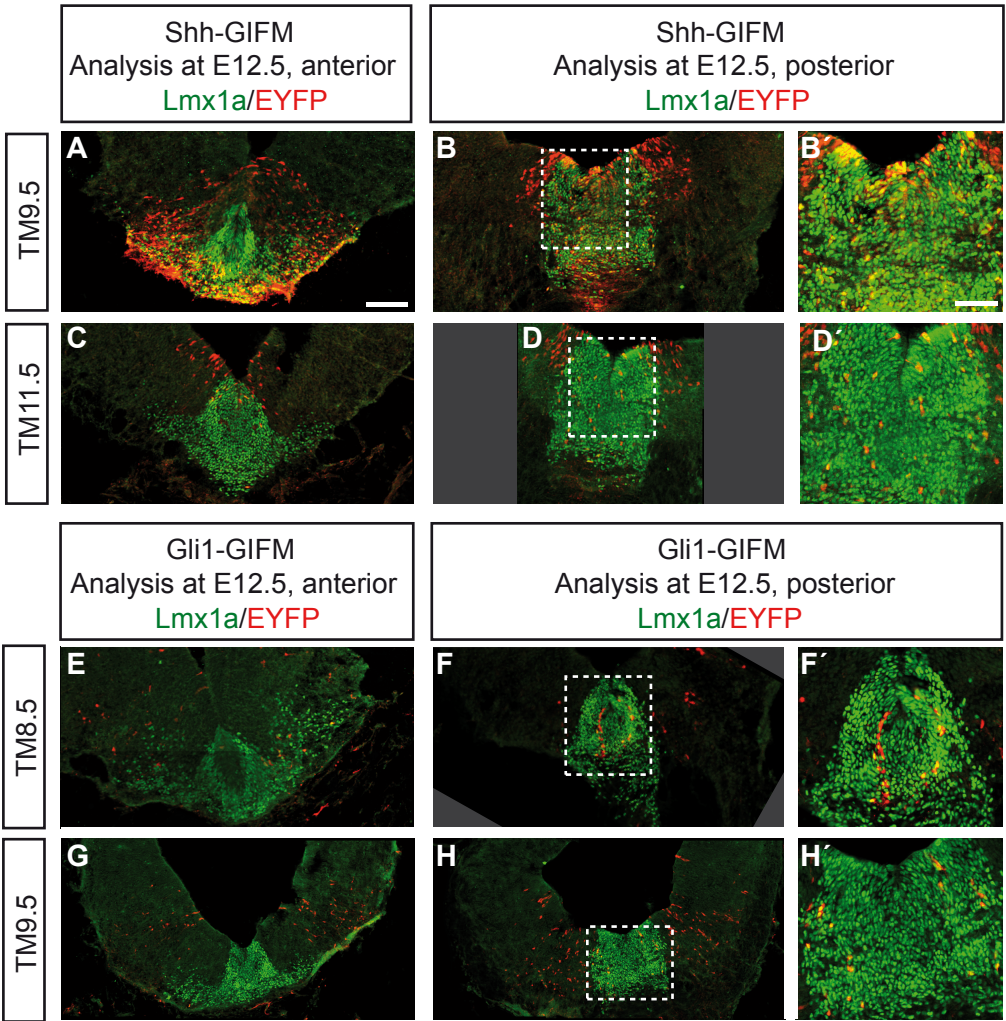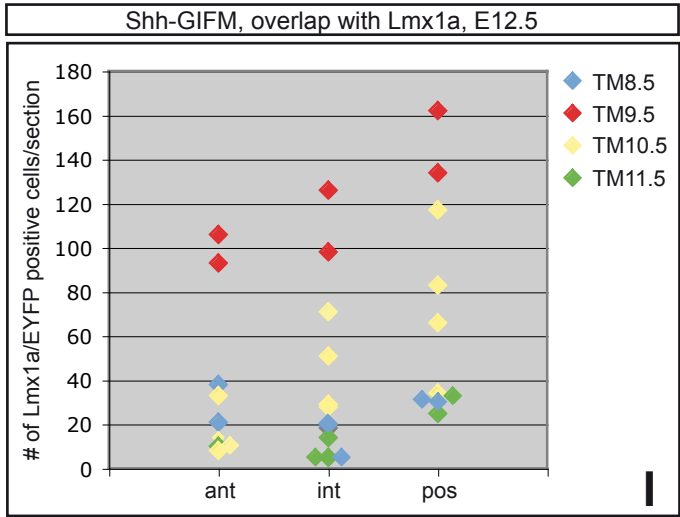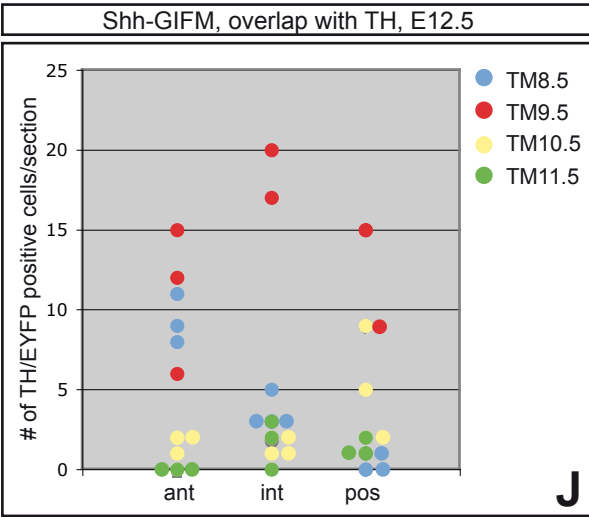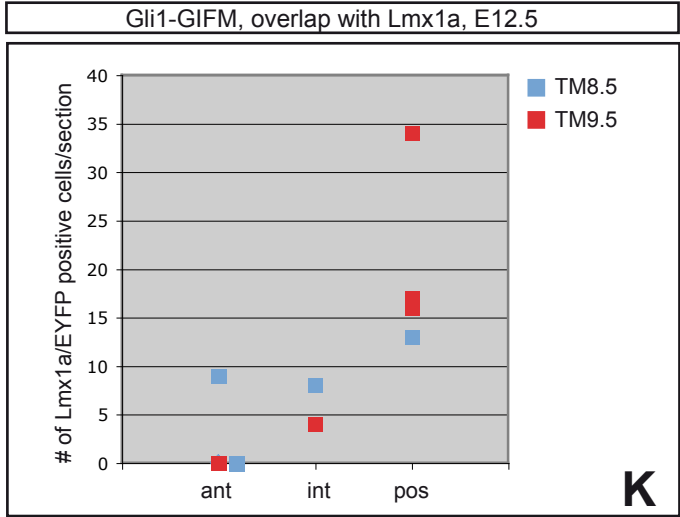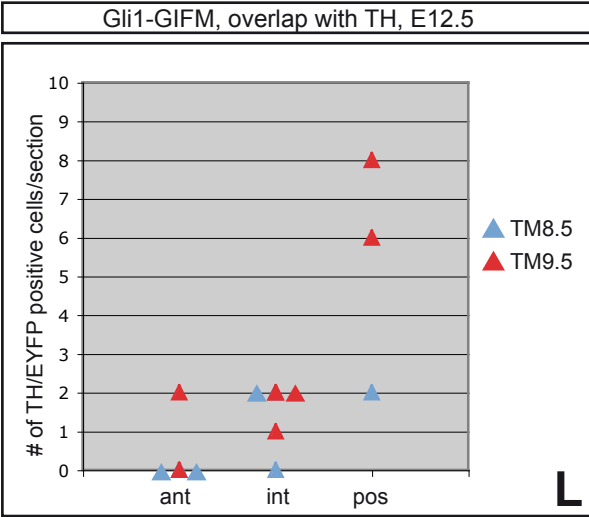

Supplement: Additional file 2 — Initial domains of cells marked with Shh- or Gli1-GIFM in comparison with Lmx1a. (A-H') TM was administered at the indicated time points and marked cells were analyzed at E12.5 on anterior and posterior coronal midbrain sections with EYFP (red) and Lmx1a (green) immunostaining. Note that on anterior sections there is no or only little overlap of Lmx1a with cells fate-mapped with Shh-GIFM at E11.5 or Gli1-GIFM at E9.5. Scale bars: (A-H) 100 μm; (B',D',F',H') 50 μm. (I-L) Number of fate-mapped cells overlapping with Lmx1a (I,K) or TH (J,L). Cells were counted on one anterior (ant), one intermediate (int) and one posterior (pos) coronal section for each E12.5 embryo (or at E11.5 for Shh-GIFM TM8.5). For one time point and one section level, each data point represents cell numbers from one embryo. Note that overlap of fate-mapped cells is much higher with Lmx1a than with TH, since Lmx1a is expressed in DA precursors and differentiated DA neurons and differentiation of DA neurons is not complete by E12.5. The trends observed at E12.5 correlate with the results of the analysis at E18.5 and in the adult brain sections: the highest contribution to DA neurons is observed with Shh-GIFM at E9.5 (I,J); cells marked with Shh-GIFM TM8.5 contribute preferentially to anterior DA neurons (J); cells marked with Shh-GIFM TM11.5 contribute preferentially to intermediate and posterior DA neurons (J); Gli1-GIFM results in less labeling than Shh-GIFM (compare (I,J) with (K,L)). [file 1749-8104-6-29-S2.PDF]
