# Supplementary material for: Temporal-spatial changes in Sonic Hedgehog expression and signaling reveal different potentials of ventral mesencephalic progenitors to populate distinct ventral midbrain nuclei
Source: Neural Dev. 2011 Jun 20;6:29. doi: 10.1186/1749-8104-6-29 (PMC3135491; doi:10.1186/1749-8104-6-29)
Supplement: Additional file 3 — Markers used to determine the location of Shh- and Gli1-derived precursors in relation to known ventral midbrain expression domains. [file 1749-8104-6-29-S3.DOC]

|  | **Expression domain** | **Neurons described to be derived from these domains** | **Method** | **Reference** |
| --- | --- | --- | --- | --- |
| **Msx1** | Most medial | DA neurons | ISH | [1] |
| **Corin** | Most medial | DA neurons | ISH | [2] |
| **Lmx1a** | Medial | DA neurons | ISH | [1] [2] |
| **Foxa2** | Medial-lateral | DA neurons | ISH | [3, 4] |
| **Nkx6-1** | Lateral | RN neurons | IF | [5, 6], |
| **Sim1** | Lateral | Glutamatergic neurons (in RN?) | ISH | [7] |
| **Nkx2-2** | Most lateral | GABAergic neurons | IF | [7-9] |

References:

1. Andersson E, Tryggvason U, Deng Q, Friling S, Alekseenko Z, Robert B, Perlmann T, Ericson J: **Identification of intrinsic determinants of midbrain dopamine neurons.** *Cell* 2006, **124:**393-405.

2. Ono Y, Nakatani T, Sakamoto Y, Mizuhara E, Minaki Y, Kumai M, Hamaguchi A, Nishimura M, Inoue Y, Hayashi H, et al: **Differences in neurogenic potential in floor plate cells along an anteroposterior location: midbrain dopaminergic neurons originate from mesencephalic floor plate cells.** *Development* 2007, **134:**3213-3225.

3. Ferri AL, Lin W, Mavromatakis YE, Wang JC, Sasaki H, Whitsett JA, Ang SL: **Foxa1 and Foxa2 regulate multiple phases of midbrain dopaminergic neuron development in a dosage-dependent manner.** *Development* 2007, **134:**2761-2769.

4. Kittappa R, Chang WW, Awatramani RB, McKay RD: **The foxa2 gene controls the birth and spontaneous degeneration of dopamine neurons in old age.** *PLoS Biol* 2007, **5:**e325.

5. Prakash N, Puelles E, Freude K, Trumbach D, Omodei D, Di Salvio M, Sussel L, Ericson J, Sander M, Simeone A, Wurst W: **Nkx6-1 controls the identity and fate of red nucleus and oculomotor neurons in the mouse midbrain.** *Development* 2009, **136:**2545-2555.

6. Moreno-Bravo JA, Perez-Balaguer A, Martinez S, Puelles E: **Dynamic expression patterns of Nkx6.1 and Nkx6.2 in the developing mes-diencephalic basal plate.** *Dev Dyn* 2010, **239:**2094-2101.

7. Nakatani T, Minaki Y, Kumai M, Ono Y: **Helt determines GABAergic over glutamatergic neuronal fate by repressing Ngn genes in the developing mesencephalon.** *Development* 2007, **134:**2783-2793.

8. Joksimovic M, Anderegg A, Roy A, Campochiaro L, Yun B, Kittappa R, McKay R, Awatramani R: **Spatiotemporally separable Shh domains in the midbrain define distinct dopaminergic progenitor pools.** *Proc Natl Acad Sci U S A* 2009.

9. Kala K, Haugas M, Lillevali K, Guimera J, Wurst W, Salminen M, Partanen J: **Gata2 is a tissue-specific post-mitotic selector gene for midbrain GABAergic neurons.** *Development* 2009, **136:**253-262.
