# Supplementary material for: Temporal-spatial changes in Sonic Hedgehog expression and signaling reveal different potentials of ventral mesencephalic progenitors to populate distinct ventral midbrain nuclei
Source: Neural Dev. 2011 Jun 20;6:29. doi: 10.1186/1749-8104-6-29 (PMC3135491; doi:10.1186/1749-8104-6-29)
Supplement: Additional file 4 — Contribution of fate-mapped cells to different regions of DA neurons along the rostral-caudal axis of the prenatal midbrain and to different subsets of DA neurons in the adult midbrain. (A,B) Number of Shh-derived (A) or Gli1-derived (B) cells contributing to different rostral-caudal regions of DA neurons at E18.5. For each animal (n ≥ 3), β-gal- and TH-co-expressing cells were counted in four regions along the rostral-caudal axis of the ventral midbrain as indicated in Figure 5B and normalized for the number of sections counted for each region. (C,D) Number of Shh-derived (C) or Gli1-derived (D) cells contributing to different subsets of DA neurons in the adult brain. For each animal (n ≥ 3), β-gal- and TH-co-expressing cells were counted in the SN, dlVTA and vmVTA as indicated in Figure 6L and normalized for the number of sections counted for each region. (E,F) Relative distribution of DA neurons in the SN, dlVTA and vmVTA (black bars) compared to the relative contribution of Shh-GIFM marked cells to the three areas. For a clearer representation, the data were split into two diagrams. The data for the fate-mapped cells (TM8.5, TM9.5, TM10.5, TM11.5) are also shown in Figure 6I. Error bars indicate standard deviation. Significance (*P < 0.05; **P < 0.01; ***P < 0.001) was determined by ANOVA and LSD post-hoc analysis (A-D) or Student's t-test (E,F). [file 1749-8104-6-29-S4.PDF]

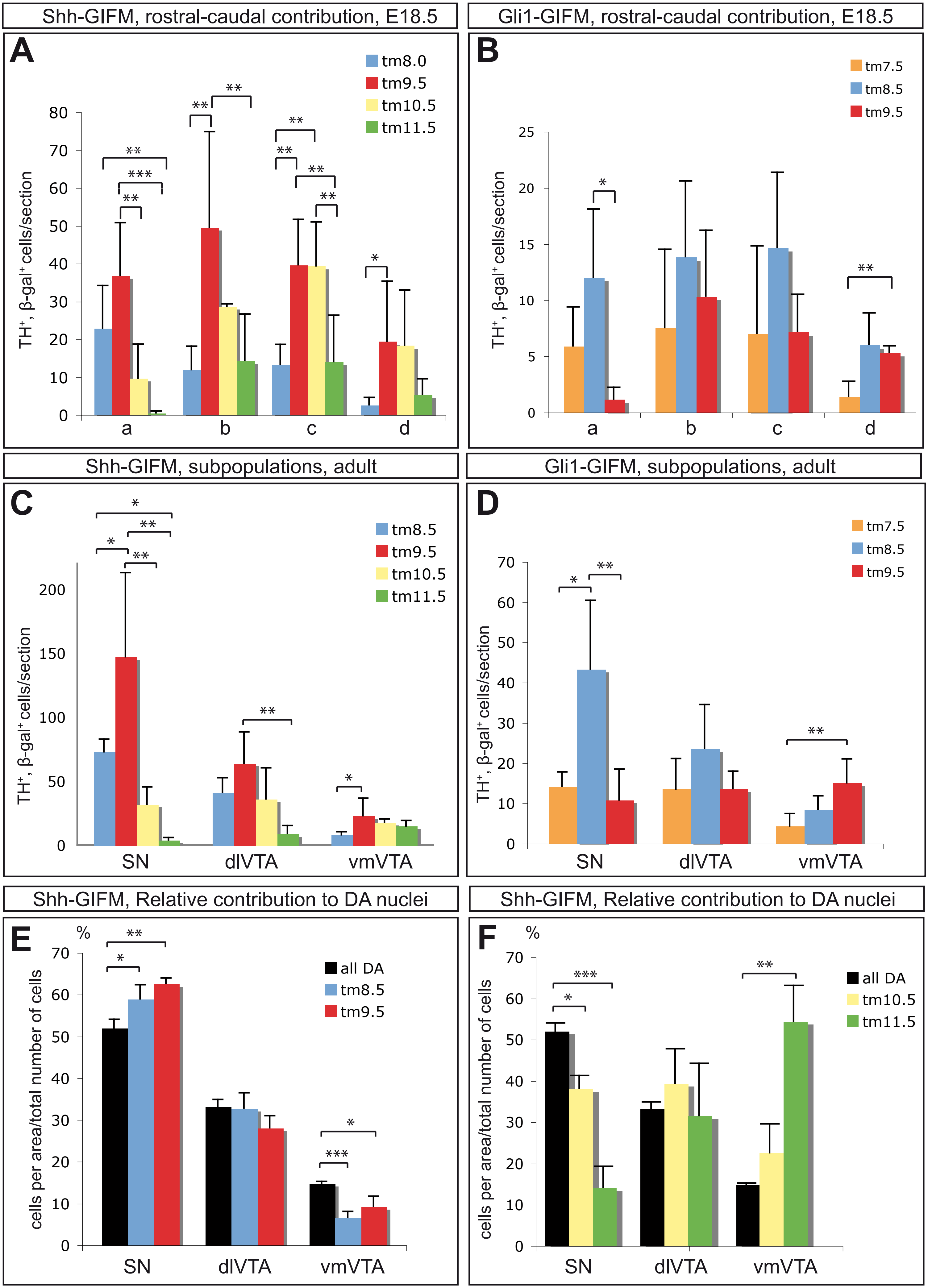

Shh-GIFM, rostral-caudal contribution, E18.5
A
tm8.0
tm9.5
tm10.5
tm11.5
TH+, β-gal+ cells/section
0
10
20
30
40
50
60
70
80
a
b
c
d
Gli1-GIFM, rostral-caudal contribution, E18.5
B
tm7.5
tm8.5
tm9.5
0
5
10
15
20
25
Shh-GIFM, subpopulations, adult
C
tm8.5
tm9.5
tm10.5
tm11.5
50
100
150
200
SN
dlVTA
vmVTA
Gli1-GIFM, subpopulations, adult
D
Shh-GIFM, Relative contribution to DA nuclei
E
%
all DA
cells per area/total number of cells
Shh-GIFM, Relative contribution to DA nuclei
F
